# Differences in excess mortality by recipient sex after liver transplant

**DOI:** 10.3389/ti.2026.16922

**Published:** 2026-10-12

**Authors:** Lauren Tal Grinspan, Xun Zhang, Mourad Dahhou, Caner Süsal, Doehler Bernd, Anette Melk, Amanda Vinson, Bethany J. Foster

**Affiliations:** 1 Recanati/Miller Transplantation Institute, Mount Sinai Hospital, New York, NY, United States; 2 Research Institute of the McGill University Health Centre, Montreal, QC, Canada; 3 UniversitatsKlinikum Heidelberg Institut fur Immunologie, Heidelberg, Germany; 4 Transplant Immunology Research Center of Excellence, Koc Universitesi, İstanbul, Türkiye; 5 Koc Universitesi Tip Fakultesi, İstanbul, Türkiye; 6 Medizinische Hochschule Hannover, Hanover, Germany; 7 Department of Medicine, Nephrology Division, Dalhousie University, Halifax, NS, Canada; 8 McGill University Department of Epidemiology Biostatistics and Occupational Health, Montreal, QC, Canada; 9 Division of Nephrology, Mcgill University Department of Pediatrics, Montreal, QC, Canada

**Keywords:** age, death, donor, gender, liver, transplant, mortality, sex

## Abstract

Identification of sex differences in mortality may prompt sex-specific management strategies. Males worldwide have higher mortality than females; therefore, we compared sex differences in *excess* mortality. We compared excess mortality in female versus male first liver transplant recipients, accounting for modifying effects of donor sex and recipient current age using relative survival models. Models were fitted separately in Scientific Registry of Transplant Recipients and CTS cohorts (1988–2019) and subsequently combined using two-stage individual patient data meta-analysis. We analyzed 218,429 patients. Within the first 3 months post-transplant, female recipients ≥25 years showed significantly higher excess mortality regardless of donor sex and female recipients 13–24 years had significantly higher excess mortality when the donor was male. Subsequent to the first 3 months, female recipients 0–12 years had significantly lower excess mortality regardless of donor sex; female recipients ≥25 years had lower excess mortality, though only significantly among those 25–44 years with a female donor. Females ≥25 years showed higher excess mortality than males in the immediate post-transplant period but, among those who survived, the first 3 months, excess mortality was similar by sex or lower in females than males. After the first 3 months post-transplant, female recipients 0–12 years showed lower excess mortality.

## Graphical Abstract

**Figure:**
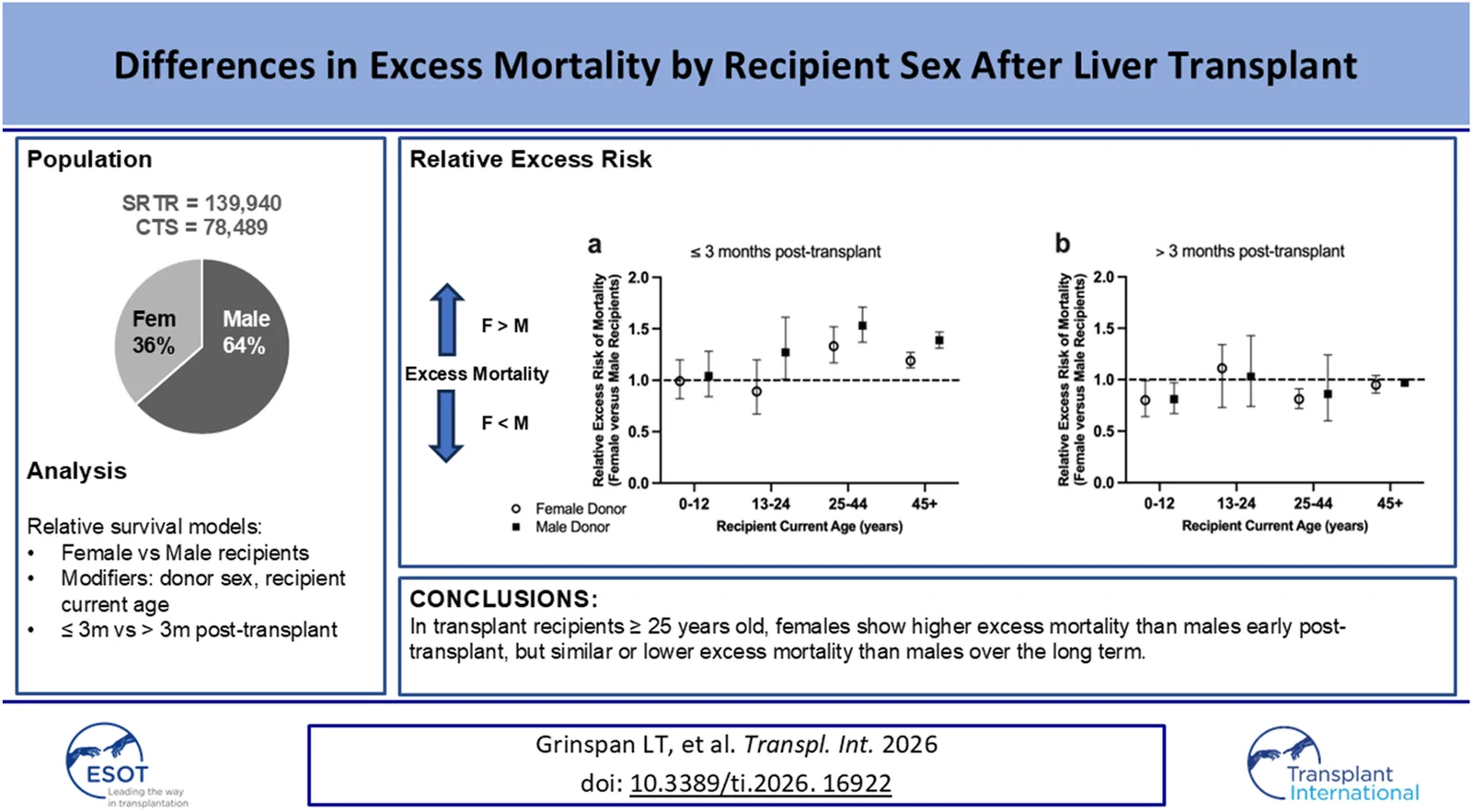

## Introduction

Both biological and social factors may contribute to sex differences in patient and graft outcomes among liver transplant recipients. An understanding of the influence of sex on graft and patient outcomes, and the mechanisms underlying these differences, may allow sex-specific management strategies such as modified immunosuppression protocols to improve graft and patient survival. Existing data on the association between recipient sex and transplant outcomes are difficult to interpret. While female recipients consistently show poorer survival than males in the early post-transplant period [1, 2], prior studies of sex differences in outcomes among those who survived the immediate post-transplant period had conflicting results [2–6].

Several studies of liver recipients who survived the early post-transplant period showed lower mortality in female than male recipients [2–5]. However, this is expected based on the known higher mortality rates in males than females worldwide [7]. Among liver transplant recipients who survived at least 5 years, the difference between observed and expected age-standardized mortality rates was substantially larger for females than males [6]. Another study that focused on recipients who survived six months-post transplant found that females were predicted to lose fewer life-years than males; the lack of confidence intervals around the predicted life years lost makes it difficult to know whether the sex difference was statistically significant [5].

It is important to recognize that sex differences in mortality risks may vary by age. Given that phenotypic expression of sexual dimorphism changes through the lifespan, it is expected that the relationship between sex and mortality risk will also change with age. No prior studies comparing mortality rates by sex in liver transplantation considered effect modification by age; the magnitude and direction of sex differences in mortality risk vary significantly by age in both kidney and heart transplantation [8, 9].

Although graft failure often leads to death, most deaths among liver transplant recipients are not caused by graft failure [10, 11]. In the early post-transplant period, only 5%–9% of deaths were reportedly due to graft failure [10, 11]; infection (28%) and cardiovascular disease (16%) were the leading causes of death [10]. Following the early post-transplant period, 80% or more of all deaths are due to causes other than graft failure [10, 11]. A study in American liver transplant recipients found variable sex differences in graft failure rates depending on recipient age and donor sex [12]. While graft failures may contribute to mortality, it is not guaranteed that sex differences in excess mortality will follow the same pattern.

We aimed to compare *excess* mortality, defined as the risk above that expected in the sex- and age-matched general population, between male and female liver transplant recipients of different ages within two periods: the first three months after transplant and time subsequent to 3 months. The comparison was undertaken in two of the largest transplant databases worldwide, the U.S. Scientific Registry of Transplant Recipients (SRTR) and the international Collaborative Transplant Study (CTS).

## Patients and methods

### Data sources and population

This was a retrospective cohort study of recipients of all ages of first liver-only deceased donor transplants recorded in the SRTR or the CTS who were transplanted January 1, 1988 to December 31, 2019 and followed until December 31, 2019. Observation ended December 31, 2019 to avoid the impact of the COVID pandemic, which led to a major increase in mortality among transplant recipients and affected male and female differently [13].

The SRTR data system includes data on all donors, waitlisted candidates, and transplant recipients in the U.S., submitted by the members of the Organ Procurement and Transplantation Network. The Health Resources and Services Administration (HRSA), US Department of Health and Human Services, provides oversight on the activities of the Organ Procurement and Transplantation Network (OPTN) and SRTR contractors. The international CTS collects data from more than 400 transplant centers in 42 countries [14]. However, only 26 countries reported data from liver transplant recipients during the observation period. We excluded countries with <100 transplants or <10 patients under observation at 10 years; only 2% of transplants reported in the CTS (eight countries) were excluded based on these criteria. Countries included in the CTS cohort are listed in the Supplementary Material. To avoid duplicates with the SRTR, recipients from the U.S. were excluded from the CTS cohort.

### Exposure and outcome definitions

#### Primary exposure

The primary exposure was recipient sex. The association between recipient sex and excess mortality is modified by donor sex and recipient current age in kidney and heart transplantation [8, 9, 15–17]. Similarly, in liver transplantation, the association between recipient sex and graft loss is modified by donor sex and recipient current age [12]. Therefore, the modifying effect of donor sex was accounted for by considering different donor-recipient sex combinations: female donor-female recipient, female donor-male recipient, male donor-female recipient, and male donor-male recipient. Additionally, a donor-recipient sex combination by recipient current age interaction term was included in all models to account for the possibility that the association between recipient sex and excess mortality may vary by age. Biological differences between males and females change with sexual development and senescence [18]. Therefore, recipient current age was a time-varying variable categorized as 0–12 (pre-pubertal), 13–24 (reproductive age), 25–44 (middle adulthood), 45–59 years (early post-reproductive age) and ≥60 years (advanced age) [15]. Relative excess risk (RER) estimates were almost identical for the age intervals 44–59 and ≥60 years; therefore, subsequent analyses combined the 45–59 and ≥60 years categories into a single interval of ≥45 years (post-reproductive age). This approach allowed us to compare excess mortality rates by sex within the different phases of life accounting for the age at which deaths occurred.

#### Primary outcome

The primary outcome was mortality with observation censored at re-transplant, end of observation, or end of the study period. Observation was limited to the first transplant because donor characteristics will change with subsequent transplants. SRTR and CTS cohorts differ in their mechanisms to capture deaths and in losses to follow-up, as previously described [8]. In the SRTR, deaths are captured by multiple mechanisms [19], including the U.S. Master Death File, and the dataset is regularly updated with deaths captured this way. As a result, even if patients are lost to follow-up, deaths will be captured in the U.S. Master Death File. Therefore, patients are assumed to be alive and under observation unless a death is reported; there are essentially no losses to follow-up for the outcome of death [19]. In contrast, deaths are reported annually to the CTS by participating centers and losses to follow-up are possible. Patients who are lost to follow-up have observation censored; deaths subsequent to censoring cannot be captured.

#### Data harmonization

New, harmonized variables were generated where differences existed between SRTR and CTS cohorts. For example, due to differences in recipient race categories between cohorts, race was categorized as White vs. Black vs. other to harmonize across cohorts.

### Statistical analysis

Analyses were conducted separately for the SRTR and CTS cohorts. Results were then combined across the cohorts, using two-stage individual patient data (IPD) meta-analysis [16, 20, 21] with a random effects model.

### Association between recipient sex and excess mortality

The association between recipient sex and excess mortality was estimated using time-dependent relative survival models with time-varying covariates [22, 23]. Relative survival models are Cox models that allow estimation of the excess risk of death among one group of individuals with the condition of interest relative to another group of individuals with the condition of interest, where excess risk is defined as the risk in individuals with the condition of interest minus the expected risk in the age-, sex-, race- (SRTR only), country-, and calendar year-matched general population [23, 24]. Note that these models account for changing calendar year-specific risks over time. Relative survival models produce RER, which are analogous to hazard ratios, and are interpreted here as the excess risk of mortality associated with liver transplant in female recipients relative to that in male recipients. Time zero was the date of transplant. Because hazards were not proportional over time, we included a time-varying time post-transplant variable (first 3 months vs. time subsequent to 3 months; cut-off point based on the time point at which the Kaplan-Meier plots showed a change in slope). This allowed separate RER to be estimated for the immediate post-transplant period (0–3 months) and long-term follow-up (>3 months).

Details of how expected mortality risk was determined for each recipient are provided in the Supplementary Material. U.S. National Vital Statistics’ general population mortality rates [25] were used to determine the expected probability of death for patients in the SRTR cohort, and country-specific data from the quality-verified [26] Human Mortality Database [27] were used to determine the expected probability of death for patients in the CTS cohort.

Initial models were unadjusted. To estimate the direct, independent association between recipient sex and excess mortality, models were adjusted for potential confounders. Acuity level at the time of transplant was not considered a potential confounder but rather a possible mediator of the relation between sex and excess mortality and was, therefore, not included. Missing data were imputed using multiple imputation methods (15 imputations) based on the fully conditional specification method; parameter estimates were pooled across the imputed datasets as per Rubin’s rule [28].

The cohort-specific RER were combined based on a weighted average of the effect sizes of the cohorts using a random effects model. Heterogeneity in RER between cohorts was assessed using Higgins *I* ^2^ and the chi-square test of heterogeneity.

### Crude excess mortality rates

For each cohort and for each time interval (first 3 months post-transplant, >3 months), we calculated crude excess mortality rates as excess deaths per 100 person-years of observation in each donor-recipient sex and age category to provide a sense of the magnitude of differences in absolute excess mortality rates by recipient sex [23]. Crude excess mortality rates were not combined across the two cohorts because there were important differences between cohorts.

### Sensitivity analysis

Because donor:recipient weight ratio was not available in the CTS, the models for SRTR were re-fitted excluding donor:recipient weight ratio to determine the impact of excluding this variable on the point estimates associated with recipient sex. Models were also re-fitted excluding recipient race since it was frequently missing in CTS.

### Comparison of graft failure by recipient sex

To better understand how graft failures may contribute to the observed sex differences in excess mortality, we also compared graft failure, defined as re-transplant or death due to graft failure, between female and male recipients of male and female donors for each recipient current age groups using time-dependent Cox models. This analysis was conducted only in the SRTR cohort; it was not possible to accurately capture deaths due to graft failure in the CTS cohort. To allow direct comparisons with the main analysis, this analysis also included an interaction by time post-transplant (first 3 months and time subsequent to 3 months post-transplant).

Data analyses were performed using Statistical Analysis System 9.4 (SAS Institute, Cary NC) and R version 4.3.0 (R Foundation for Statistical Computing, Vienna, Austria. http://www.R-project.org/). The study was approved by the McGill University Health Center Research Ethics Board (2020-6264) and conducted in accordance with the principles of the Declaration of Helsinki. The need for consent was waived.

## Results

### Patient characteristics

Table 1 provides an overview of the characteristics of each cohort. A total of 218,429 recipients met the inclusion criteria, namely 139,940 in the SRTR (36.4% female) and 78,489 in the CTS (36.0% female). There were 49,501 deaths in the SRTR (35.4%) over 5.2 [interquartile range (IQR) 1.6–10.7] years of follow-up and 24,009 in the CTS (30.6%) over 4.0 [IQR 1.0–9.5] years of follow-up.

**TABLE 1:** Overview of patient characteristics (n, %). (unit of analysis here is the patient).

Variable	SRTR	CTS
Patients	139,940	78,489
Censoring Death Retransplant	49,501 (35.4) 9,981 (7.1)	24,009 (30.6) 7,718 (9.8)
Follow up (years) - median (IQR)	5.2 [1.6–10.7]	4.0 [1.0–9.5]
Age at transplant (years) - median, IQR	53 [44–60]	52 [41–59]
Recipient race White Black Other Missing (%)	119,684 (85.5) 12,574 (9.0) 7,682 (5.5) 0	39,675 (93.1) 649 (1.5) 2,269 (5.3) 35,896 (45.7)
Donor-recipient sex Female donor → female recipient Female donor → male recipient Male donor → female recipient Male donor → male recipient	23,866 (17.1) 31,815 (22.7) 27,075 (19.3) 57,184 (40.9	15,026 (19.1) 19,136 (24.4) 12,779 (16.3) 31,548 (40.2)
Primary disease Congenital/biliary atresia Alcohol-associated liver disease Liver tumors Metabolic liver disease Fulminant liver failure Autoimmune conditions Hepatitis C Others	6,781 (4.8) 21,364 (15.3) 18,698 (13.4) 5,005 (3.6) 8,175 (5.8) 17,145 (12.3) 36,329 (26.0) 26,443 (18.9)	4,339 (5.5) 12,093 (15.4) 10,639 (13.6) 3,064 (3.9) 6,141 (7.8) 10,296 (13.1) 20,062 (25.6) 11,855 (15.1)
Donor age (years) - median, IQR	38 [22–52]	47 [30–60]
Transplant year 1988–1994 1995–1999 2000–2004 2005–2009 2010–2014 2015–2019	15,982 (10.4) 17,857 (12.8) 21,629 (15.5) 25,836 (18.5) 26,233 (18.7) 32,403 (23.1)	8,493 (10.8) 11,717 (14.9) 14,449 (18.4) 15,642 (19.9) 15,496 (19.7) 12.692 (16.2)
Cold ischemia time (hours) <8 8-<12 ≥12 Missing (%)	83,100 (63.1) 36,177 (27.5) 12,477 (9.5) 8,186 (5.8)	25,608 (38.3) 30,134 (45.1) 11,056 (16.6) 11,691 (14.9)
Donor-recipient weight ratio (%) ≥0.9 <0.9 Missing (%)	77,631 (58.2) 55,747 (41.8) 6,562 (4.7)	Not available Not available 78,489 (100)
Graft size Whole Reduced	135,132 (96.6) 4,808 (3.4)	71,529 (91.1) 6,960 (8.9)
Donor CMV Positive Negative Missing (%)	86,339 (62.1) 52,600 (37.9) 1,001 (0.7)	30,782 (53.9) 26,332 (46.1) 21,375 (27.2)
Donor cause of death Cerebrovascular accident Other Missing (%)	50,121 (35.8) 89,708 (64.2) 111 (0.1)	41,302 (57.4) 30,601 (42.6) 6,586 (8.4)
Donor hypertension Yes No Missing (%)	38,463 (30.7) 86,872 (69.3) 14,605 (10.4	7,495 (10.4) 64,916 (89.6) 6,078 (7.7)
Donor donation after cardiac death Yes No Missing (%)	4,236 (3.0) 135,704 (97.0) 0 (0.0)	869 (1.2) 72,717 (98.8) 4,903 (6.2)

A higher proportion of patients in the SRTR (9.0%) than CTS (1.5%) were Black; however recipient race was missing for 45.7% in the CTS (0% in SRTR). The median age of donors was younger in SRTR (38 [IQR 22–52] years) than CTS (47 [IQR 30–60] years). Additionally, the cold ischemia time was lower in the SRTR cohort compared with the CTS cohort. Partial livers were more frequently used in CTS compared with SRTR (8.9% vs. 3.4%). Donor hypertension was more common in SRTR than CTS (30.7% vs. 10.4%) and donor cause of death was more frequently CVA, in CTS, than SRTR (57.4% vs. 35.8%).

Table 2 (first 3 months post-transplant) and Table 3 (>3 months post-transplant) summarize the composition of the observed experience within each age interval for male and female recipients of male and female donors, combined across the two cohorts. Cohort specific data are shown in the Supplementary Tables S1–S4.

**TABLE 2:** Composition of the observed experience of female and male recipients by donor sex and recipient current age during the firs**t** 3 months post-transplant combined across SRTR and CTS (proportion of person years**)**.

​	Female donor								Male donor							
Recipient current age	0–12 years		13–24 years		25–44 years		45+ years		0–12 years		13–24 years		25–44 years		45+ years	
Recipient sex	Female	Male	Female	Male	Female	Male	Female	Male	Female	Male	Female	Male	Female	Male	Female	Male
Person years of observation	721	708	442	330	1628	1745	6048	8889	1033	1043	528	488	1687	3255	5821	15,819
Deaths	220	228	141	109	509	428	1831	2427	282	291	167	120	559	754	1817	3991
Age at transplant																
0–12 (%)	100.0	100.0	0.0	0.0	0.0	0.0	0.0	0.0	100.0	100.0	0.0	0.0	0.0	0.0	0.0	0.0
13–24 (%)	0.0	0.0	100.0	100.0	0.0	0.0	0.0	0.0	0.0	0.0	100.0	100.0	0.0	0.0	0.0	0.0
25–44 (%)	0.0	0.0	0.0	0.0	100.0	100.0	0.0	0.0	0.0	0.0	0.0	0.0	100.0	100.0	0.0	0.0
45 + y (%)	0.0	0.0	0.0	0.0	0.0	0.0	100.0	100.0	0.0	0.0	0.0	0.0	0.0	0.0	100.0	100.0
Recipient race																
White (%)	75.8	79.5	78.0	82.4	84.4	85.9	87.7	88.5	75.5	78.8	78.7	81.6	83.3	88.5	87.9	90.1
Black (%)	15.6	12.4	16.5	13.5	10.6	7.8	6.7	5.5	15.2	12.6	16.1	13.2	11.9	6.4	7.1	5.3
Others (%)	8.6	8.1	5.5	4.1	5.0	6.2	5.6	6.0	9.3	8.6	5.3	5.2	4.7	5.0	5.0	4.6
Missing (%)	0.1	0.2	0.1	0.2	0.2	0.2	0.2	0.2	0.1	0.2	0.1	0.1	0.1	0.2	0.1	0.2
Primary disease																
Congenital/biliary atresia (%)	59.4	48.0	10.8	14.5	3.1	1.5	1.1	0.3	58.8	47.4	11.9	14.2	3.2	1.3	1.2	0.3
Alcohol -associated liver disease (%)	0.0	0.0	0.1	0.6	11.6	18.9	11.6	19.9	0.1	0.1	0.2	0.5	11.7	20.0	11.6	20.4
Liver tumors (%)	5.6	7.5	3.9	3.8	5.1	6.2	10.8	20.3	5.9	8.8	3.4	4.8	4.4	5.4	9.9	19.5
Metabolic liver disease (%)	11.7	17.1	12.8	13.0	3.8	4.8	1.5	2.1	11.6	17.1	15.6	12.5	4.4	4.3	1.8	2.2
Fulminant liver failure (%)	9.2	13.0	30.2	21.7	17.2	7.6	5.1	2.5	9.5	12.5	27.2	21.2	19.1	6.3	5.8	2.2
Autoimmune conditions (%)	2.3	2.2	22.5	24.1	28.1	16.7	23.9	6.0	2.6	1.9	22.3	23.8	26.4	16.0	21.5	5.7
Hepatitis C (%)	0.8	1.1	2.7	5.7	12.4	26.8	24.8	33.2	0.8	1.1	2.4	4.1	13.0	28.7	25.4	33.5
Others (%)	10.9	11.2	17.0	16.6	18.8	17.6	21.2	15.6	10.7	11.0	17.0	18.9	17.8	18.0	22.7	16.1
Donor age (years)																
0–5 (%)	42.0	38.7	0.8	0.8	0.1	0.0	0.1	0.0	40.7	38.1	1.4	0.9	0.3	0.1	0.2	0.0
6–12 (%)	17.6	17.4	7.1	8.0	2.6	1.0	2.0	0.6	18.6	18.3	10.3	9.8	4.1	1.0	3.4	0.6
13–17 (%)	11.2	11.5	14.1	13.5	6.5	5.2	4.8	3.2	13.1	13.8	22.2	18.8	12.1	8.0	9.1	5.1
18–34 (%)	14.4	16.2	26.1	25.1	24.5	22.9	20.1	19.0	19.9	21.7	38.4	38.2	40.8	40.0	35.5	31.9
35–49 (%)	11.5	12.3	29.5	30.4	31.1	33.1	26.9	28.7	6.3	6.7	16.9	20.1	21.9	25.6	22.2	25.3
50–59 (%)	2.6	3.1	14.3	15.9	19.9	21.2	21.5	22.9	1.2	1.2	7.3	7.8	12.7	15.4	14.7	18.3
≥60 (%)	0.7	0.8	8.1	6.3	15.2	16.5	24.7	25.6	0.2	0.2	3.6	4.5	8.2	10.0	15.0	18.9
Cold ischemia (hours)																
<8 (%)	49.2	49.8	53.0	53.4	53.6	51.2	60.4	61.5	50.1	50.5	53.9	51.3	53.9	50.6	61.2	60.8
8-<12 (%)	35.1	35.9	32.6	31.7	31.2	33.8	28.5	28.8	35.2	34.2	32.5	32.1	31.0	32.3	27.5	29.0
≥12 (%)	15.7	14.2	14.5	14.9	15.2	15.0	11.1	9.7	14.7	15.3	13.7	16.6	15.1	17.1	11.3	10.2
Missing (%)	14.7	16.0	16.6	14.6	16.2	14.8	12.5	12.2	16.2	16.0	14.6	16.0	13.6	14.2	11.3	12.1
Transplant year																
1988–1994 (%)	18.5	16.5	17.7	13.9	17.1	14.6	9.0	5.0	18.8	18.9	20.7	17.7	21.3	19.3	11.7	6.9
1995–1999 (%)	15.7	14.7	15.6	16.3	17.7	19.6	12.9	10.2	14.2	15.1	16.6	17.2	19.0	20.2	13.9	10.7
2000–2004 (%)	15.7	15.7	18.2	18.7	17.6	18.9	16.4	16.3	17.2	14.2	16.4	17.0	14.7	18.1	16.0	16.4
2005–2009 (%)	18.3	18.7	17.0	16.4	16.7	15.9	19.1	21.9	16.1	17.1	15.7	17.8	15.0	15.2	17.7	21.0
2010–2014 (%)	17.2	17.9	16.2	17.2	14.7	15.2	20.4	23.4	16.3	17.4	15.4	15.0	13.4	11.6	19.2	21.6
2015–2019 (%)	14.7	16.5	15.3	17.5	16.2	15.8	22.2	23.3	17.4	17.3	15.1	15.4	16.5	15.6	21.5	23.4
Donor:recipient weight ratio																
≥0.9 (%)	80.9	81.3	60.8	60.8	57.7	43.1	57.5	43.5	83.2	83.1	72.1	72.8	66.8	57.7	67.1	58.5
<0.9 (%)	19.1	18.7	39.2	39.2	42.3	56.9	42.5	56.5	16.8	16.9	27.9	27.2	33.2	42.3	32.9	41.5
Missing (%)	7.9	7.7	8.1	6.3	7.6	5.8	4.0	2.7	7.5	7.5	8.6	6.7	7.4	6.5	4.1	3.3
Graft size																
Whole (%)	61.3	58.4	93.3	94.1	96.8	98.2	97.6	98.8	57.6	56.3	91.4	92.2	95.8	97.8	95.9	98.3
Reduced (%)	38.7	41.6	6.7	5.9	3.2	1.8	2.4	1.2	42.4	43.7	8.6	7.8	4.2	2.2	4.1	1.7

Because the unit of analysis was person-time, rather than person, the characteristics presented are weighted by a factor derived from the number of person-years of observation and number of events. For example, 59.4% of the person-years contributed by female recipients between 0 and 12 years of a female donor were by people with congenital liver disease as the primary disease.

There were sex differences in the distribution of primary disease, particularly among those 25 years or older, with a greater proportion of the person-years of males than females contributed by patients with alcohol-associated liver disease or hepatitis C and a greater proportion of the person-years of females than males contributed by patients with autoimmune liver disease or fulminant hepatic failure. Among those 25 years or older, a greater proportion of the person-years of females than males were contributed by patients with donor-to-recipient weight ratio ≥0.9. These sex differences were consistent regardless of donor sex.

**TABLE 3:** Composition of the observed experience of female and male recipients by donor sex and recipient current age after the firs**t** 3 months post-transplant combined across SRTR and CTS (proportion of person years).

​	Female donor								Male donor							
Recipient current age	0–12 years		13–24 years		25–44 years		45+ years		0–12 years		13–24 years		25–44 years		45+ years	
Recipient sex	Female	Male	Female	Male	Female	Male	Female	Male	Female	Male	Female	Male	Female	Male	Female	Male
Person years of observation	17,609	16,621	15,399	13,358	36,685	31,414	189,326	239,287	24,927	24,284	20,233	19,914	39,731	58,066	189,875	452,016
Deaths	151	185	240	181	950	1053	8636	12,637	215	272	291	262	1064	1820	8578	23,056
Age at transplant																
0–12 (%)	100.0	100.0	55.4	59.5	2.7	3.1	0.0	0.0	100.0	100.0	57.2	60.6	3.8	2.5	0.0	0.0
13–24 (%)	0.0	0.0	44.6	40.5	19.4	13.6	0.1	0.0	0.0	0.0	42.8	39.4	21.1	12.2	0.1	0.0
25–44 (%)	0.0	0.0	0.0	0.0	77.9	83.3	12.9	10.8	0.0	0.0	0.0	0.0	75.1	85.3	13.4	12.0
45 + y (%)	0.0	0.0	0.0	0.0	0.0	0.0	87.1	89.1	0.0	0.0	0.0	0.0	0.0	0.0	86.5	88.0
Recipient race																
White (%)	75.4	79.8	77.9	84.1	83.3	84.8	88.6	88.7	74.9	78.7	78.8	82.1	83.5	87.0	88.7	91.1
Black (%)	16.4	12.3	16.0	10.9	11.9	9.0	6.2	5.0	16.0	13.4	15.1	12.2	12.3	7.9	6.7	4.6
Others (%)	8.2	7.9	6.1	5.0	4.8	6.3	5.2	6.4	9.1	7.9	6.1	5.8	4.2	5.0	4.5	4.3
Missing (%)	0.1	0.1	0.1	0.1	0.1	0.2	0.1	0.2	0.1	0.1	0.1	0.1	0.1	0.1	0.1	0.1
Primary disease																
Congenital/biliary atresia (%)	66.1	54.8	36.3	36.5	5.6	5.0	1.2	0.3	65.6	53.8	38.8	35.9	6.7	3.9	1.4	0.3
Alcohol-associated liver disease (%)	0.0	0.1	0.1	0.1	6.1	12.3	9.9	19.2	0.0	0.1	0.0	0.1	5.8	13.8	9.8	19.8
Liver tumors (%)	4.2	5.9	3.1	3.3	3.7	4.5	7.3	15.7	4.9	7.1	3.3	4.2	3.2	4.3	6.6	14.3
Metabolic liver disease (%)	11.0	16.4	15.4	16.4	6.9	7.5	1.7	2.6	9.7	16.9	15.1	16.5	8.2	7.6	2.0	2.8
Fulminant liver failure (%)	7.3	11.1	17.8	16.3	23.2	11.8	6.8	3.0	7.9	10.7	16.6	16.4	24.5	9.6	7.4	2.7
Autoimmune conditions (%)	1.2	1.2	10.7	11.0	27.4	21.4	31.3	7.8	1.7	1.2	10.7	10.6	25.2	20.4	28.7	8.1
Hepatitis C (%)	0.4	0.7	1.3	2.5	7.5	18.9	21.6	34.9	0.7	0.9	1.4	2.5	7.8	20.7	22.6	35.1
Others (%)	9.8	9.7	15.2	13.8	19.7	18.6	20.2	16.5	9.4	9.5	14.1	13.7	18.5	19.8	21.5	16.9
Donor age (years)																
0–5 (%)	49.1	46.4	24.2	24.8	1.4	1.2	0.1	0.0	47.0	43.9	24.2	23.0	1.8	1.0	0.2	0.0
6–12 (%)	16.8	16.5	18.0	18.7	4.5	2.9	2.7	0.8	17.3	17.3	19.5	21.8	6.7	3.0	4.6	0.7
13–17 (%)	10.6	10.0	14.4	12.4	8.7	6.7	6.5	4.7	11.5	12.6	18.4	16.6	15.6	10.2	12.5	7.3
18–34 (%)	11.9	13.4	18.0	17.7	26.7	25.1	22.7	21.0	17.8	19.4	24.9	25.3	41.0	42.5	39.5	36.7
35–49 (%)	9.3	10.3	15.8	16.7	30.1	32.7	29.4	30.3	5.1	5.8	8.7	9.5	19.3	23.7	20.8	25.4
50–59 (%)	1.9	2.9	6.7	7.1	17.5	18.7	20.2	22.2	1.1	0.8	3.2	2.6	10.0	12.6	12.2	15.8
≥60 (%)	0.6	0.5	3.0	2.5	11.2	12.6	18.4	20.9	0.1	0.2	1.1	1.2	5.6	7.0	10.3	14.1
Cold ischemia (hours)																
<8 (%)	47.1	46.7	42.6	45.3	48.6	48.0	52.6	55.0	46.9	48.2	44.8	45.0	48.1	46.6	52.8	53.4
8-<12 (%)	36.4	37.3	38.3	35.8	33.5	35.3	32.3	32.6	37.0	34.9	36.7	34.3	33.7	33.6	31.6	32.9
≥12 (%)	16.5	16.0	19.1	18.9	17.9	16.8	15.1	12.4	16.0	16.9	18.5	20.8	18.2	19.8	15.7	13.7
Missing (%)	14.0	16.0	15.6	16.3	16.6	15.6	13.0	12.4	15.9	16.8	16.0	17.1	14.8	15.2	12.6	12.3
Transplant year																
1988–1994 (%)	20.2	19.7	25.4	25.3	23.1	19.5	16.3	8.2	21.2	21.3	27.9	27.3	28.3	25.0	19.9	11.9
1995–1999 (%)	18.9	16.3	25.1	25.0	22.4	22.1	20.1	16.7	16.7	18.2	23.9	25.5	23.0	22.5	21.9	17.9
2000–2004 (%)	19.0	19.3	21.7	22.8	20.5	21.3	22.6	23.6	21.6	17.7	22.5	20.4	16.9	19.4	20.5	23.4
2005–2009 (%)	22.2	23.2	15.3	14.5	17.7	17.5	20.6	25.7	19.9	21.1	13.6	15.3	16.3	16.8	18.7	23.8
2010–2014 (%)	14.9	16.1	9.2	9.0	11.5	13.7	14.7	18.8	14.7	15.9	8.9	8.7	10.5	10.5	13.6	16.6
2015–2019 (%)	4.8	5.4	3.3	3.4	4.9	5.9	5.6	7.0	5.9	5.7	3.2	2.9	5.1	5.9	5.4	6.5
Donor:recipient weight ratio																
≥0.9 (%)	83.8	83.7	68.7	71.1	59.6	47.4	56.6	41.3	85.4	85.0	74.4	76.5	68.7	60.9	66.1	56.9
<0.9 (%)	16.2	16.3	31.3	28.9	40.4	52.6	43.4	58.7	14.6	15.0	25.6	23.5	31.3	39.1	33.9	43.1
Missing (%)	8.8	8.6	10.6	10.0	10.4	7.9	6.6	3.9	8.6	9.0	9.4	10.5	9.6	8.7	6.6	4.9
Graft size																
Whole (%)	60.7	58.8	83.2	82.3	96.6	97.9	97.6	98.7	58.2	56.4	78.6	77.3	95.3	97.0	96.0	98.3
Reduced (%)	39.3	41.2	16.8	17.7	3.4	2.1	2.4	1.3	41.8	43.6	21.4	22.7	4.7	3.0	4.0	1.7

Because the unit of analysis was person-time, rather than person, the characteristics presented are weighted by a factor derived from the number of person-years of observation and number of events. For example, 66.0% of the person-years contributed by female recipients between 0 and 12 years of a female donor were by people with congenital liver disease as the primary disease.

There were sex differences in the distribution of primary disease, particularly among those 25 years or older, with a greater proportion of the person-years of males than females contributed by patients with alcohol-associated liver disease or hepatitis C and a greater proportion of the person-years of females than males contributed by patients with autoimmune liver disease or fulminant hepatic failure. Among those 25 years or older, a greater proportion of the person-years of females than males were contributed by patients with donor-to-recipient weight ratio ≥0.9. These sex differences were consistent regardless of donor sex.

Medical condition at the time of transplant was captured in the SRTR with a variable indicating whether the patient was hospitalized in the intensive care unit and whether the patient was on life support at the time of transplant. Among those over 12 years old, females were consistently more likely to be in the intensive care unit and on life support than males (Supplementary Table S5). In the CTS, condition at transplant was captured with a variable indicating urgency level (super-urgent, urgent, or normal). Among those over 12 years old, females were consistently more likely to be classified as super-urgent or urgent than males. In both SRTR and CTS, among those 12 years or younger, differences by sex in level of acuity were very small and in the opposite direction to older recipients, with a very slightly higher proportion of males than females showing high acuity.

### Relative excess mortality risk by recipient sex

Forest plots (Figure 1) show the adjusted RER of mortality for female compared with male recipients by donor sex (a) in the first 3 months post-transplant and (b) after 3 months post-transplant. The RER are shown for each cohort separately, along with the combined RER from the IPD meta-analysis. Results of unadjusted analyses are shown in Supplementary Figure S1.

**FIGURE 1:**
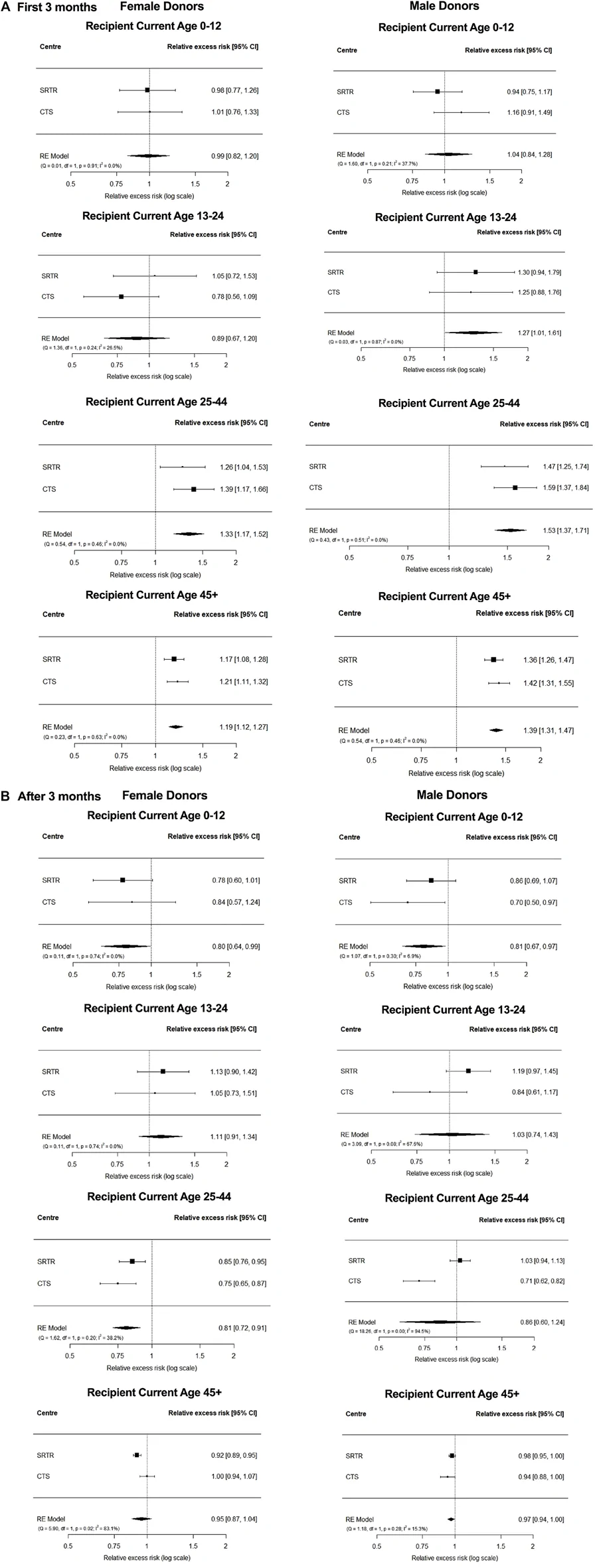
Multivariable IPD-meta-analysis results for excess mortality in female versus male recipients of female and male donors **(A)** during the first three months post-transplant and **(B)** after the first three months post-transplant. Forest plots show the relative excess risks (RER) of mortality in female versus male recipients in each age interval. RER are shown separately for the SRTR and CTS cohorts, as well as the pooled RER from the meta-analysis. RER >1.00 indicate higher excess mortality in female than male recipients; RER <1.00 indicate lower excess mortality in female than male recipients. Models were adjusted for recipient race (White, Black, or Other), primary disease (congenital/biliary atresia, alcohol-related liver disease, liver tumors, metabolic liver disease, fulminant liver failure, autoimmune conditions, or hepatitis **C)**, donor age (linear and quadratic), transplant year (continuous linear), cold ischemia time (continuous linear), graft size (whole or reduced), and donor:recipient weight ratio (a measure of donor-recipient body size match or mismatch categorized as <0.9, and ≥0.9; not available for CTS). RER are analogous to hazard ratios and interpreted as excess risk of mortality associated with transplant in female recipients relative to that in males.

Figure 2 summarizes the pooled adjusted RER in female versus male recipients in the first 3 months post-transplant and after the first 3 months post-transplant in the four recipient age intervals. In the first 3 months, excess mortality was significantly higher in female than male recipients ≥25 years regardless of donor sex. Among 13–24-year-olds, female recipients showed significantly higher excess mortality than male recipients only when the donor was male.

**FIGURE 2:**
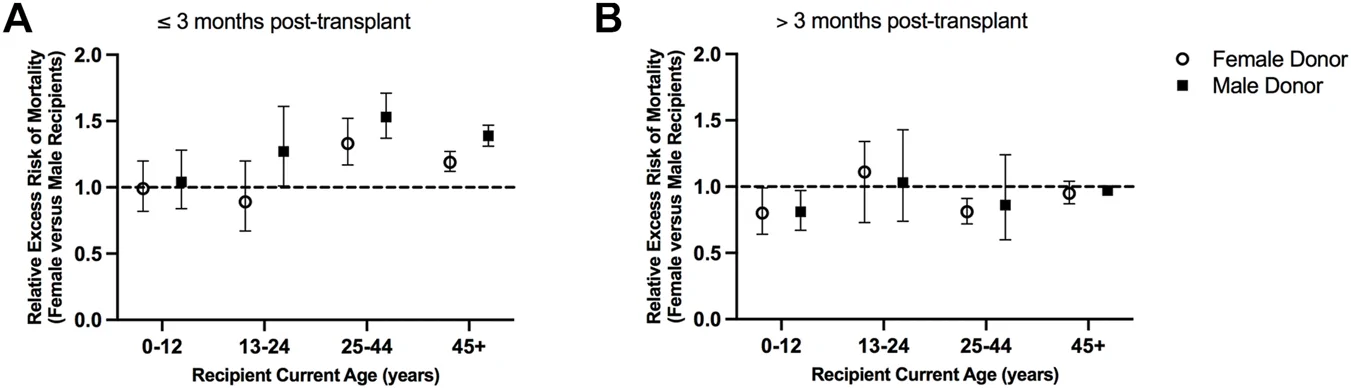
Summary of pooled adjusted relative excess risks of mortality in female versus male recipients of female and male donors **(A)** during the first three months post-transplant and **(B)** after the first three months post-transplant. RER >1.00 indicate higher excess mortality in female than male recipients; RER <1.00 indicate lower excess mortality in female than male recipients. Models were adjusted for transplant year (continuous linear), cold ischemia time (continuous linear), graft size (whole or reduced), and donor:recipient weight ratio (a measure of donor-recipient body size match or mismatch categorized as <0.9, and ≥0.9; not available for CTS). Relative excess risks (RER) are analogous to hazard ratios and interpreted as excess risk of mortality associated with transplant in female recipients relative to that in males.

After 3 months post-transplant, female recipients 0–12 years showed significantly lower excess mortality than male recipients of the same age regardless of donor sex. There were no clear differences in excess mortality by recipient sex among 13–24-year-olds regardless of donor sex. Among those ≥25 years old, female recipients had lower excess mortality than males, though the difference was only statistically significant among 25–44-year-old recipients of a female donor. There was some heterogeneity between the cohorts.

### Between cohort heterogeneity

There was very little between-cohort heterogeneity in the RER of mortality for female versus male liver recipients within the first 3 months post-transplant. However, after the first 3 months, there was more substantial between-cohort heterogeneity, especially among recipients aged 13–24 years (*I* ^*2*^ = 67.6%) and 25–44 years (*I* ^*2*^ = 94.5%) who had a male donor and recipients ≥45 years (*I* ^*2*^ = 83.1%) who had a female donor.

### Sensitivity analysis

Results of models in the SRTR cohort including and excluding donor-recipient weight ratio, shown in Supplementary Table S6, and of models in both SRTR and CTS including and excluding recipient race (Supplementary Table S7) were very similar, with point estimates differing by less than 10%.

### Comparison of graft failure by recipient sex

The results of analyses comparing graft failure rates by recipient sex in the SRTR cohort are shown in Supplementary Figure S2 side-by-side with SRTR-specific sex differences in excess mortality rates.

#### First three months

In the setting of a female donor, graft failure rates were significantly lower in female than male recipients aged 25–44 years (HR 0.81, 95%CI 0.68, 0.96) and ≥45 years (HR 0.73, 95%CI 0.60–0.89); there were no differences by recipient sex among those <25 years. In the setting of a male donor, female recipients <25 years had higher graft failure rates than male recipients of the same age, but differences were not statistically significant; there were no clear differences in graft failure rates by recipient sex among those 25 years and older.

#### After the first three months

When the donor was female, there were no convincing differences in graft failure rates by recipient sex at any age. When the donor was male, graft failure rates were higher for female than male recipients 13–24 years (HR 1.19, 95%CI 1.07–1.33) and lower for female than male recipients ≥45 years (HR 0.94, 95%CI 0.90–0.98).

### Crude excess mortality rates

As shown in Figure 3, crude excess mortality rates were lower in the SRTR than the CTS within the first three months post-transplant. In contrast, beyond the first three months, the crude excess mortality rates for CTS were slightly lower than for SRTR. The absolute differences in crude excess mortality rates by recipient sex were small for younger recipients but were substantial among older recipients in the first three months post-transplant (Figure 3). In the first three months post-transplant, crude excess mortality rates were between 5.96 per 100 person-years lower in females than males (13–24-year-old recipients of a female donor in CTS) and 18.3 per 100 person-years higher in females than males (25–44-year-old recipients of a male donor in CTS). Crude excess mortality rates were consistently higher in females than males 25 years and older in both cohorts. After the first three months post-transplant, crude excess mortality rates were between 0.93 per 100 person-years lower in females than males (25–44-year-old recipients of a male donor in CTS) and 0.3 per 100 person-years higher in females than males (13–24-year-old recipients in SRTR).

**FIGURE 3:**
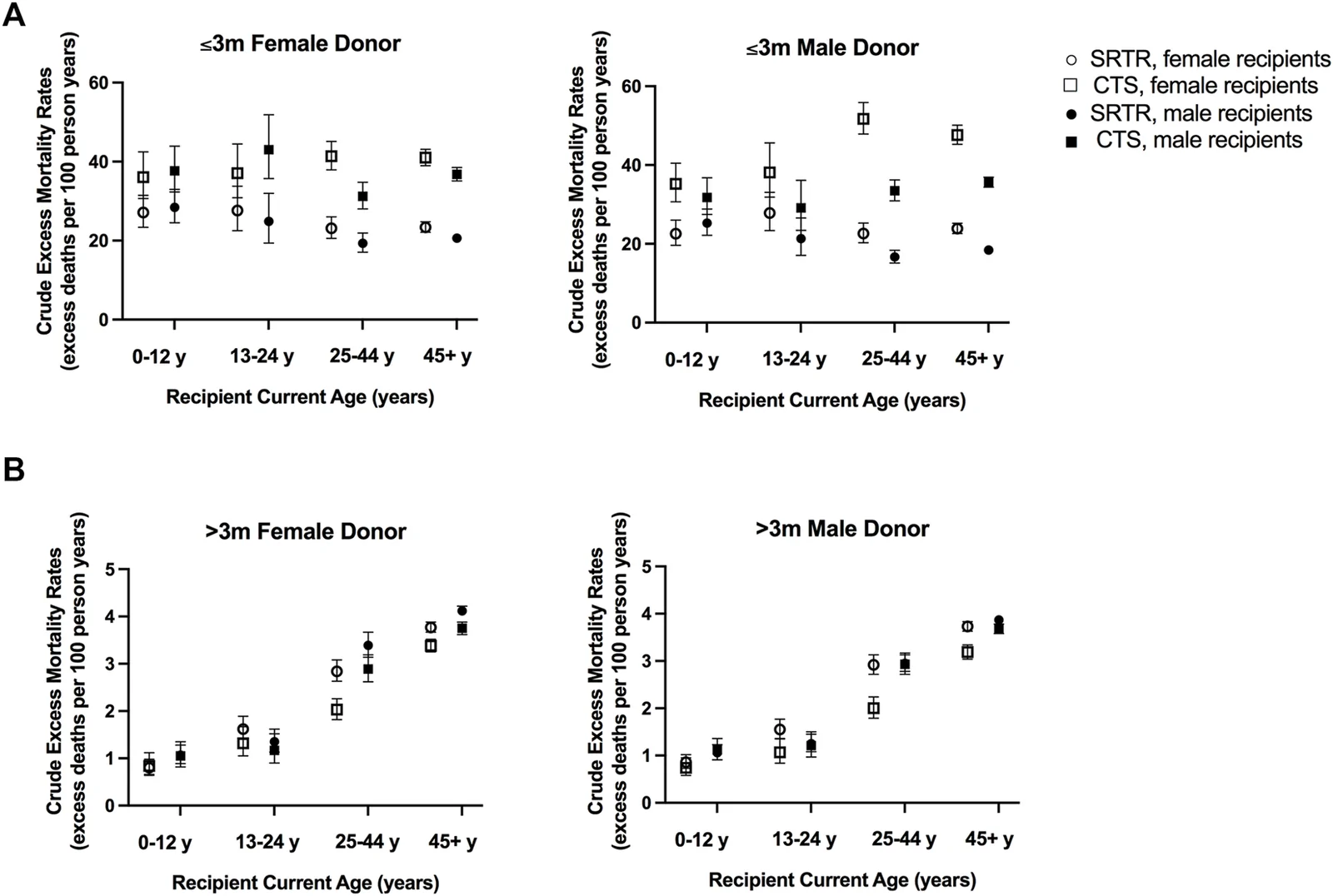
Crude excess mortality rates for female and male recipients **(A)** during the first three months post-transplant and **(B)** after the first three months post-transplant by recipient current age and donor sex. Crude excess mortality rates (excess deaths per 100 person-years of observation) of female and male recipients separately for SRTR and CTS cohorts are plotted by donor sex during the first three months post-transplant and after the first three months post-transplant.

Recipient cause of death was analyzed for each sex-age category by donor sex in SRTR (Supplementary Table S8a) and CTS (Supplementary Table S8b).

## Discussion

This study used IPD meta-analysis to compare excess mortality between female and male liver transplant recipients in two of the largest databases worldwide. Deaths after liver transplant may be related to graft failure (rejection or other causes) or to causes unrelated to graft function (such as infection or malignancy).

Consistent with prior studies [1, 2] in the early post-transplant period, we found substantially and significantly higher excess mortality in female than male liver transplant recipients over 24 years old, regardless of donor sex, and in female than male recipients 13–24 years when the donor was male. There were no differences in excess mortality by sex among children 0–12 years old. The higher excess mortality in females than males in the first three months post-transplant likely reflects the fact that females must be sicker than males to receive a transplant. The MELD score, used to prioritize liver allocation for those >12 years old in the US from 2002 to 2023 and in Eurotransplant from 2006 to 2025 [29], underestimates the risk of mortality for women [30–33]. Implementation in 2023 in the U.S. of MELD 3.0 and in 2025 in Europe of reMELD-Na updated scores to more accurately predict waitlist mortality, which may improve this disparity in excess mortality [34–36]. Prior to the implementation of the MELD score, allocation protocols may also have disadvantaged women, resulting in women dying on the waiting list more frequently than men and being transplanted in worse condition [37]. A recent study comparing excess mortality by sex among waitlisted American liver candidates found significantly higher excess mortality among females than males only for candidates 13–24- and ≥60-years-old [38]; this study did not compare condition at transplant. Among those over 12 years, a greater proportion of females than males in our SRTR cohort were on life support and in intensive care at the time of transplant and a greater proportion of females than males in our CTS cohort were classified as super-urgent or urgent; there were no such differences among those <13 years old.

During observation subsequent to the first three months, excess mortality was lower for female than male recipients of all ages except 13–24 years, though differences were only statistically significant for recipients 25–44 years when the donor was female and recipients 0–12 years regardless of donor sex. Among those 13–24 years, there were no significant differences in excess mortality by sex. Among those over 12 years old, the lower excess mortality in females than males or lack of difference in excess mortality by sex in the period after the first three months may represent selection bias wherein only the healthiest, most resilient women survive to three months post-transplant. A higher risk for malignancy in male than female liver transplant recipients may also play a role in this observation [39]. Higher graft failure rates in female than male recipients 13–24-years-old may also contribute to the observed pattern of sex differences in excess mortality rates, with a higher risk of graft failure-related mortality counterbalanced by a lower risk of death from other causes due to selection bias. The reasons for the significantly lower excess mortality in females than males under 13 years who survived at least three months post-transplant is not clear and requires further investigation; selection bias is unlikely in this age group.

A 2022 Spanish study of almost 16,000 liver recipients over 16-years-old showed significantly higher mortality in females than males in the first year post-transplant but slightly better 10-year survival in females than males. The leading causes of death were infection (23%) and primary disease recurrence (21%) in women and infection (19%) and malignancy (15%) in men [2]. Causes of death were not reported by early versus late deaths. A study of 872 pediatric liver transplant recipients who had at least one year of follow-up after transplant reported malignancy (20%), infection (15%), and post-transplant lymphoproliferative disorder (15%) as the most common causes of death; primary disease recurrence was extremely uncommon [40].

Unlike in kidney [8, 16] and heart transplantation [9, 17], it is unclear whether sex differences in graft loss rates contribute to the observed differences in excess mortality by recipient sex among liver transplant recipients. In the first three months post-transplant, graft failure rates were significantly *lower* for female than male recipients ≥25 years when the donor was female and not different for female versus male recipients when the donor was male, while SRTR-specific excess mortality rates were significantly *higher* for female than male recipients; this suggests that sex differences in excess mortality in the first three months for those ≥25 years are related to factors other than graft failure. Among those 13–24 years with a male donor, graft failure rates were higher for female than male recipients, suggesting that graft losses may contribute to the higher excess mortality. After the first three months, graft failure rates were higher among adolescent and young adult female than male recipients of a male donor and lower among female than male recipients 45 years and older, suggesting that sex differences in graft failure rates may contribute to the observed sex differences in excess mortality in these age groups.

Sex disparities in access to the transplantation waitlist may also contribute to sex differences in excess mortality post-transplant, though data specific to liver transplantation are sparse [41–43]. There is evidence in kidney and heart transplantation that women are less likely than men to be referred and waitlisted and that women who are waitlisted have fewer comorbidities than men [41, 44]. This selection will bias toward lower excess mortality in females than males after transplant.

The substantially higher crude excess mortality rates in the CTS compared with the SRTR cohort within the first three months is notable. This may reflect an overall poorer condition at the time of transplant in the CTS. Among those recipients for whom a MELD score was available (only 34% of recipients in CTS >12 years old and transplanted in 2006 or later versus almost 100% of those in SRTR >12 years old and transplanted in 2003 or later), scores were higher in CTS than SRTR. Reasons for the higher excess mortality in the primarily European CTS cohort than in the SRTR cohort require additional study.

### Limitations

This is the largest study to examine outcomes after liver transplant and the first to compare *excess* mortality by recipient sex with adjustment for potential confounders and accounting for effect modification by recipient age. However, there are limitations. Substantial heterogeneity between cohorts in the period subsequent to the first three months for some age categories (i.e. recipients aged 25–44 years with a male donor I^2^ = 94.5%) suggests that some combined RER should be interpreted with caution. In addition, confounding due to unmeasured variables cannot be excluded. Furthermore, race was poorly captured in the CTS cohort. If race were an important confounder in the relation between recipient sex and excess mortality, this may introduce bias. However, the sensitivity analysis suggests that race was not acting as a confounder here.

Whereas graft status at death is recorded well in SRTR, this is not reliably captured in CTS, making it impossible to distinguish with confidence between death due to graft loss and death with graft function in the CTS. This limitation adds to the challenge of identifying reasons for the observed sex differences in excess mortality. Although there was potential for incomplete capture of deaths, it is unlikely that any incomplete capture of deaths differed by sex; therefore, this would be unlikely to lead to bias. An SRTR study that assessed the impact of missing deaths showed no significant differences in extension of survival time between male and female recipients [19].

Differences in mechanisms to capture deaths and in losses to follow-up between the SRTR and CTS are an additional limitation. Although these differences are unlikely to lead to bias in the observed relation between sex and excess mortality, they may result in differences across cohorts in absolute mortality rates and crude excess mortality rates. Because crude excess mortality rates depend on both observed mortality rates in the transplant cohort and expected mortality (based on general population rates), they may be particularly difficult to compare across cohorts. For example, higher excess mortality rates for CTS patients in the first three months post-transplant compared with the SRTR may have been driven by both relatively high observed mortality rates in CTS and somewhat lower expected mortality rates. Due to these limitations, we avoided combining absolute excess mortality rates across cohorts.

## Conclusion

In the early post-transplant period, female liver transplant recipients 25 years and older show higher excess mortality than males of the same age, regardless of donor sex. Poorer condition of female patients at the time of transplant may be responsible for these differences. After the first three months, female recipients 0–12 years showed significantly lower excess mortality than males regardless of donor sex, which requires further investigation. Among recipients >12 years, sex differences in excess mortality after the first three months were less consistent. Females ≥25 years showed a tendency toward lower excess mortality than males of the same age. Possible reasons for this observation include sex bias in access to transplantation (with healthier females than males getting access), survival bias, wherein only the healthiest females survive the first three months, and higher risk of malignancy in males. Importantly, multiple factors may contribute to sex differences—or even to lack of sex differences—in excess mortality. Sex differences in graft failure rates and in deaths unrelated to graft failure in the same or opposite directions will combine to determine excess mortality differences. Further study is needed to determine the causes of the observed patterns.

## Data Availability

The data analyzed in this study is subject to the following licenses/restrictions: data are available exclusively from the Scientific Registry of Transplant Recipients and CTS data are not publicly available. Requests to access these datasets should be directed to srtr@srtr.org.
