# Supplementary material for: Differences in excess mortality by recipient sex after liver transplant
Source: Transpl Int. 2026 Oct 12;39:16922. doi: 10.3389/ti.2026.16922 (PMC13697205; doi:10.3389/ti.2026.16922)
Supplement: Supplementary file 1 [file Supplementaryfile1.docx]

**Supplement**

**Countries included in the CTS cohort**

This study included data from centers in Australia, Austria, Belgium, Canada, Columbia, France, Germany, Hungary, Ireland, Israel, Italy, Netherlands, Slovenia, Spain, Sweden, Switzerland, Turkey, and the United Kingdom.

**Detailed Methods**

In order to determine expected mortality risk for each recipient as they were followed over time, a dynamic dataset that updated age and calendar year of observation was created, accounting for their changing age-related risk and the changing calendar year-related risk [1]. To accomplish this, each patient’s observation was split into multiple 1-year intervals following the date of transplant. Each patient’s expected probability of death within each 1-year interval was determined based on the publicly available age- (matched based on the following categories: <1, 1–4, 5–9 years, and 5-year age intervals thereafter), race- (U.S. only), sex-, and calendar year-specific general population mortality rates in the relevant geographical area. We assumed a constant hazard within each interval. The hazard function at any given time since transplant was modeled as the sum of the expected hazard and the excess hazard associated with transplant [2]**.**

**Supplemental Table S1:** Composition of the contrasted experience by recipient sex and age for recipients of female and male donors within the first 3 months (SRTR only)

|  | **Female Donor** | | | | | | | | **Male Donor** | | | | | | | |
| --- | --- | --- | --- | --- | --- | --- | --- | --- | --- | --- | --- | --- | --- | --- | --- | --- |
| **Recipient Current Age** | **0-12 years** | | **13-24 years** | | **25-44 years** | | **45+ years** | | **0-12 years** | | **13-24 years** | | **25-44 years** | | **45+ years** | |
| **Recipient sex** | **Female** | **Male** | **Female** | **Male** | **Female** | **Male** | **Female** | **Male** | **Female** | **Male** | **Female** | **Male** | **Female** | **Male** | **Female** | **Male** |
| **Person-years of observation** | 464 | 439 | 246 | 184 | 912 | 1017 | 3905 | 5752 | 666 | 658 | 334 | 290 | 1085 | 2036 | 4190 | 10492 |
| **Deaths** | 127 | 126 | 68 | 46 | 212 | 199 | 942 | 1250 | 152 | 168 | 93 | 62 | 247 | 344 | 1031 | 2049 |
| **Expected number of deaths** | 1.1 | 1.1 | 0.1 | 0.2 | 1.1 | 2.3 | 28.5 | 62.7 | 1.5 | 1.6 | 0.1 | 0.3 | 1.3 | 4.8 | 29.9 | 113.3 |
| **Excess mortality rate (deaths per 1000 person-years)** | 125.9 | 124.9 | 67.9 | 45.8 | 210.9 | 196.7 | 913.5 | 1187.3 | 150.5 | 166.4 | 92.9 | 61.7 | 245.7 | 339.2 | 1001.1 | 1935.7 |
| **Age at transplant (years)** |  |  |  |  |  |  |  |  |  |  |  |  |  |  |  |  |
| 0-12 (%) | 100.0 | 100.0 | 0.0 | 0.0 | 0.0 | 0.0 | 0.0 | 0.0 | 100.0 | 100.0 | 0.0 | 0.0 | 0.0 | 0.0 | 0.0 | 0.0 |
| 13-24 (%) | 0.0 | 0.0 | 100.0 | 100.0 | 0.0 | 0.0 | 0.0 | 0.0 | 0.0 | 0.0 | 100.0 | 100.0 | 0.0 | 0.0 | 0.0 | 0.0 |
| 25- 44 (%) | 0.0 | 0.0 | 0.0 | 0.0 | 100.0 | 100.0 | 0.0 | 0.0 | 0.0 | 0.0 | 0.0 | 0.0 | 100.0 | 100.0 | 0.0 | 0.0 |
| 45+y (%) | 0.0 | 0.0 | 0.0 | 0.0 | 0.0 | 0.0 | 100.0 | 100.0 | 0.0 | 0.0 | 0.0 | 0.0 | 0.0 | 0.0 | 100.0 | 100.0 |
| **Recipient race** |  |  |  |  |  |  |  |  |  |  |  |  |  |  |  |  |
| White (%) | 72.4 | 76.9 | 72.3 | 76.4 | 80.7 | 83.5 | 85.4 | 87.1 | 72.0 | 76.3 | 73.5 | 76.7 | 80.2 | 87.0 | 86.4 | 88.9 |
| Black (%) | 19.8 | 15.1 | 23.5 | 18.8 | 14.1 | 9.6 | 8.5 | 6.7 | 19.3 | 15.9 | 21.7 | 17.9 | 15.2 | 8.0 | 8.5 | 6.4 |
| Others (%) | 7.8 | 7.9 | 4.2 | 4.8 | 5.2 | 6.8 | 6.1 | 6.2 | 8.7 | 7.9 | 4.9 | 5.4 | 4.6 | 4.9 | 5.1 | 4.6 |
| Missing (%) | 72.4 | 76.9 | 72.3 | 76.4 | 80.7 | 83.5 | 85.4 | 87.1 | 72.0 | 76.3 | 73.5 | 76.7 | 80.2 | 87.0 | 86.4 | 88.9 |
| **Primary disease** |  |  |  |  |  |  |  |  |  |  |  |  |  |  |  |  |
| Congenital/biliary atresia (%) | 62.5 | 50.1 | 11.3 | 15.8 | 2.2 | 1.2 | 0.4 | 0.2 | 61.0 | 47.3 | 12.6 | 15.9 | 1.9 | 1.2 | 0.4 | 0.2 |
| Alcohol Associated Liver Disease (%) | 0.0 | 0.0 | 0.1 | 0.8 | 15.0 | 22.1 | 10.7 | 19.1 | 0.1 | 0.0 | 0.3 | 0.7 | 14.3 | 22.8 | 11.1 | 19.4 |
| Liver tumors (%) | 5.9 | 8.6 | 4.3 | 3.5 | 4.3 | 5.1 | 11.6 | 20.1 | 6.4 | 9.6 | 3.8 | 4.7 | 3.5 | 4.2 | 10.6 | 18.8 |
| Metabolic liver disease (%) | 12.0 | 17.9 | 12.5 | 11.9 | 2.9 | 4.9 | 1.5 | 2.1 | 11.6 | 18.5 | 15.1 | 11.6 | 3.6 | 3.9 | 1.7 | 2.3 |
| Fulminant liver failure (%) | 8.6 | 13.2 | 27.4 | 22.0 | 15.6 | 6.3 | 4.3 | 2.5 | 9.6 | 14.6 | 25.7 | 21.9 | 18.0 | 5.7 | 5.1 | 2.4 |
| Autoimmune conditions (%) | 2.4 | 1.9 | 25.8 | 26.3 | 29.2 | 15.6 | 21.7 | 5.8 | 2.6 | 1.6 | 25.3 | 25.3 | 27.8 | 16.1 | 20.2 | 5.8 |
| Hepatitis C (%) | 0.3 | 0.1 | 3.0 | 4.6 | 12.8 | 27.6 | 24.7 | 33.1 | 0.1 | 0.2 | 1.4 | 2.6 | 13.5 | 28.4 | 25.2 | 33.7 |
| Others (%) | 8.4 | 8.2 | 15.6 | 15.1 | 18.0 | 17.2 | 25.1 | 17.2 | 8.5 | 8.2 | 15.8 | 17.4 | 17.4 | 17.6 | 25.7 | 17.5 |
| **Donor Age (years)** |  |  |  |  |  |  |  |  |  |  |  |  |  |  |  |  |
| 0-5 (%) | 55.1 | 51.9 | 1.0 | 0.9 | 0.1 | 0.0 | 0.1 | 0.0 | 51.7 | 48.9 | 1.3 | 0.7 | 0.4 | 0.0 | 0.2 | 0.0 |
| 6-12 (%) | 17.8 | 17.7 | 9.3 | 9.3 | 3.5 | 1.4 | 2.5 | 0.8 | 17.4 | 17.7 | 10.6 | 11.4 | 4.8 | 1.2 | 4.0 | 0.7 |
| 13-17 (%) | 11.0 | 11.3 | 17.5 | 16.1 | 7.9 | 6.4 | 5.7 | 4.0 | 13.7 | 14.2 | 25.2 | 21.2 | 13.8 | 9.5 | 10.0 | 6.1 |
| 18-34 (%) | 9.4 | 12.3 | 29.1 | 28.1 | 27.0 | 26.2 | 22.9 | 22.8 | 14.1 | 16.3 | 41.2 | 41.6 | 42.9 | 43.8 | 37.8 | 36.7 |
| 35-49 (%) | 5.4 | 5.2 | 25.5 | 27.3 | 30.4 | 33.0 | 27.2 | 30.9 | 2.3 | 2.5 | 13.6 | 17.6 | 20.6 | 24.6 | 21.9 | 25.9 |
| 50-59 (%) | 1.1 | 1.4 | 11.4 | 13.6 | 19.0 | 20.2 | 21.1 | 22.9 | 0.6 | 0.3 | 5.3 | 4.9 | 11.1 | 13.7 | 13.9 | 17.3 |
| ≥ 60 (%) | 0.2 | 0.2 | 6.2 | 4.7 | 12.2 | 12.9 | 20.5 | 18.7 | 0.1 | 0.0 | 2.9 | 2.6 | 6.4 | 7.1 | 12.1 | 13.3 |
| **Cold ischemia (hours)** |  |  |  |  |  |  |  |  |  |  |  |  |  |  |  |  |
| <8 (%) | 53.3 | 54.6 | 59.2 | 62.2 | 59.5 | 56.9 | 66.4 | 67.3 | 54.7 | 55.4 | 59.2 | 57.1 | 58.1 | 54.8 | 65.7 | 66.0 |
| 8-<12 (%) | 33.7 | 33.9 | 29.5 | 27.8 | 28.0 | 31.2 | 25.5 | 26.0 | 32.9 | 32.4 | 30.7 | 30.2 | 29.6 | 30.8 | 25.3 | 26.4 |
| ≥12 (%) | 13.0 | 11.5 | 11.3 | 9.9 | 12.5 | 11.9 | 8.1 | 6.7 | 12.4 | 12.2 | 10.2 | 12.7 | 12.3 | 14.4 | 8.9 | 7.7 |
| Missing (%) | 8.0 | 6.9 | 6.6 | 7.2 | 6.9 | 7.2 | 5.3 | 4.8 | 7.8 | 7.1 | 7.8 | 8.4 | 6.6 | 6.6 | 5.5 | 5.0 |
| **Transplant year** |  |  |  |  |  |  |  |  |  |  |  |  |  |  |  |  |
| 1988-1994 (%) | 20.0 | 19.3 | 18.8 | 16.2 | 20.2 | 15.6 | 9.2 | 4.9 | 19.7 | 20.0 | 20.5 | 18.6 | 22.2 | 21.5 | 11.1 | 7.0 |
| 1995-1999 (%) | 15.7 | 14.2 | 16.0 | 14.8 | 18.4 | 20.8 | 12.3 | 9.5 | 13.3 | 13.9 | 14.5 | 14.0 | 18.0 | 20.1 | 13.0 | 9.8 |
| 2000-2004 (%) | 15.3 | 14.6 | 17.1 | 18.4 | 15.6 | 17.9 | 15.5 | 15.6 | 16.6 | 12.4 | 15.0 | 16.4 | 13.6 | 16.0 | 14.3 | 15.6 |
| 2005-2009 (%) | 17.8 | 17.2 | 17.2 | 16.6 | 14.6 | 13.6 | 18.4 | 21.4 | 14.8 | 16.4 | 16.7 | 18.4 | 13.9 | 13.6 | 17.7 | 20.9 |
| 2010-2014 (%) | 16.3 | 17.4 | 14.8 | 15.1 | 13.0 | 14.2 | 19.8 | 22.8 | 16.3 | 17.9 | 16.5 | 15.4 | 12.5 | 10.7 | 19.8 | 21.2 |
| 2015-2019 (%) | 14.9 | 17.2 | 16.1 | 19.0 | 18.1 | 17.9 | 24.8 | 25.8 | 19.2 | 19.4 | 16.9 | 17.3 | 19.8 | 18.1 | 24.2 | 25.5 |
| **Donor :recipient weight ratio*** |  |  |  |  |  |  |  |  |  |  |  |  |  |  |  |  |
| >=0.9 (%) | 80.9 | 81.3 | 60.8 | 60.8 | 57.7 | 43.1 | 57.5 | 43.5 | 83.2 | 83.1 | 72.1 | 72.8 | 66.8 | 57.7 | 67.1 | 58.5 |
| <0.9 (%) | 19.1 | 18.7 | 39.2 | 39.2 | 42.3 | 56.9 | 42.5 | 56.5 | 16.8 | 16.9 | 27.9 | 27.2 | 33.2 | 42.3 | 32.9 | 41.5 |
| Missing (%) | 7.9 | 7.7 | 8.1 | 6.3 | 7.6 | 5.8 | 4.0 | 2.7 | 7.5 | 7.5 | 8.6 | 6.7 | 7.4 | 6.5 | 4.1 | 3.3 |
| **Graft size** |  |  |  |  |  |  |  |  |  |  |  |  |  |  |  |  |
| Whole (%) | 74.2 | 72.6 | 96.4 | 98.4 | 98.6 | 99.3 | 98.6 | 99.4 | 68.7 | 67.0 | 94.3 | 95.6 | 98.1 | 99.0 | 97.1 | 99.0 |
| Reduced (%) | 25.8 | 27.4 | 3.6 | 1.6 | 1.4 | 0.7 | 1.4 | 0.6 | 31.3 | 33.0 | 5.7 | 4.4 | 1.9 | 1.0 | 2.9 | 1.0 |

Because the unit of analysis was person-time, rather than person, the characteristics presented are weighted by a factor derived from the number of person-years of observation and number of events. For example, 62.5% of the person-years contributed by female recipients between 0–12 years who had a female donor were by people with congenital disease/ biliary atresia as the primary disease.

*SRTR only

**Supplemental Table S2:** Composition of the contrasted experience by recipient sex and age for recipients of female and male donors after the first 3 months (SRTR only)

|  | **Female Donor** | | | | | | | | **Male Donor** | | | | | | | |
| --- | --- | --- | --- | --- | --- | --- | --- | --- | --- | --- | --- | --- | --- | --- | --- | --- |
| **Recipient Current Age** | **0-12 years** | | **13-24 years** | | **25-44 years** | | **45+ years** | | **0-12 years** | | **13-24 years** | | **25-44 years** | | **45+ years** | |
| **Recipient sex** | **Female** | **Male** | **Female** | **Male** | **Female** | **Male** | **Female** | **Male** | **Female** | **Male** | **Female** | **Male** | **Female** | **Male** | **Female** | **Male** |
| **Person-years of observation** | 12109 | 10933 | 10300 | 8696 | 20821 | 17971 | 125031 | 159994 | 16776 | 16007 | 14040 | 13283 | 25133 | 36033 | 137682 | 311690 |
| **Deaths** | 102 | 122 | 171 | 125 | 616 | 649 | 6030 | 8864 | 152 | 177 | 223 | 178 | 762 | 1143 | 6576 | 16450 |
| **Expected number of deaths** | 4.9 | 5.3 | 3.7 | 7.7 | 24.2 | 39.3 | 1312.9 | 2264.4 | 7.1 | 7.6 | 4.9 | 11.9 | 29.1 | 81.4 | 1442.2 | 4372.6 |
| **Excess mortality rate** | 97.1 | 116.7 | 167.3 | 117.3 | 591.8 | 609.7 | 4717.1 | 6599.6 | 144.9 | 169.4 | 218.1 | 166.1 | 732.9 | 1061.6 | 5133.8 | 12077.4 |
| **Age at transplant (years)** |  |  |  |  |  |  |  |  |  |  |  |  |  |  |  |  |
| 0-12 (%) | 100.0 | 100.0 | 60.6 | 64.1 | 3.8 | 4.3 | 0.0 | 0.0 | 100.0 | 100.0 | 59.7 | 62.8 | 4.8 | 3.1 | 0.0 | 0.0 |
| 13-24 (%) | 0.0 | 0.0 | 39.4 | 35.9 | 19.6 | 13.6 | 0.1 | 0.0 | 0.0 | 0.0 | 40.3 | 37.2 | 20.5 | 12.1 | 0.1 | 0.0 |
| 25- 44 (%) | 0.0 | 0.0 | 0.0 | 0.0 | 76.6 | 82.1 | 12.3 | 10.5 | 0.0 | 0.0 | 0.0 | 0.0 | 74.7 | 84.8 | 12.8 | 11.9 |
| 45+y (%) | 0.0 | 0.0 | 0.0 | 0.0 | 0.0 | 0.0 | 87.6 | 89.5 | 0.0 | 0.0 | 0.0 | 0.0 | 0.0 | 0.0 | 87.1 | 88.0 |
| **Recipient race** |  |  |  |  |  |  |  |  |  |  |  |  |  |  |  |  |
| White (%) | 72.2 | 77.2 | 74.2 | 80.9 | 79.0 | 81.4 | 86.3 | 87.3 | 71.1 | 76.1 | 75.0 | 79.4 | 79.9 | 84.8 | 87.1 | 90.0 |
| Black (%) | 20.1 | 14.6 | 20.7 | 13.9 | 16.5 | 11.5 | 7.9 | 6.0 | 19.9 | 16.5 | 19.2 | 15.6 | 16.2 | 10.2 | 8.2 | 5.6 |
| Others (%) | 7.7 | 8.1 | 5.2 | 5.2 | 4.5 | 7.2 | 5.7 | 6.6 | 9.0 | 7.3 | 5.8 | 5.0 | 3.9 | 5.1 | 4.7 | 4.4 |
| Missing (%) | 72.2 | 77.2 | 74.2 | 80.9 | 79.0 | 81.4 | 86.3 | 87.3 | 71.1 | 76.1 | 75.0 | 79.4 | 79.9 | 84.8 | 87.1 | 90.0 |
| **Primary disease** |  |  |  |  |  |  |  |  |  |  |  |  |  |  |  |  |
| Congenital/biliary atresia (%) | 68.4 | 56.6 | 41.1 | 41.0 | 5.9 | 5.7 | 0.6 | 0.3 | 67.8 | 53.8 | 42.1 | 37.8 | 6.8 | 4.7 | 0.6 | 0.3 |
| Alcohol Associated Liver Disease (%) | 0.0 | 0.0 | 0.0 | 0.2 | 7.7 | 14.3 | 9.2 | 18.6 | 0.1 | 0.0 | 0.1 | 0.1 | 7.0 | 15.8 | 9.5 | 18.8 |
| Liver tumors (%) | 4.4 | 6.7 | 3.1 | 3.1 | 3.3 | 3.7 | 7.8 | 15.0 | 5.3 | 8.0 | 3.7 | 4.2 | 2.6 | 3.4 | 6.9 | 13.1 |
| Metabolic liver disease (%) | 11.7 | 17.5 | 15.2 | 16.5 | 6.2 | 7.7 | 1.8 | 2.6 | 9.6 | 18.2 | 13.7 | 17.2 | 7.5 | 7.2 | 2.1 | 2.9 |
| Fulminant liver failure (%) | 7.0 | 11.4 | 15.6 | 16.1 | 21.9 | 9.9 | 6.1 | 3.2 | 8.0 | 12.2 | 15.5 | 17.5 | 22.7 | 8.6 | 6.9 | 3.0 |
| Autoimmune conditions (%) | 1.2 | 0.9 | 11.5 | 10.7 | 30.1 | 22.5 | 29.6 | 7.9 | 1.5 | 0.8 | 11.8 | 10.8 | 28.5 | 21.6 | 27.7 | 8.5 |
| Hepatitis C (%) | 0.1 | 0.0 | 0.9 | 1.5 | 6.8 | 17.9 | 21.9 | 35.0 | 0.1 | 0.1 | 0.6 | 1.3 | 7.4 | 19.4 | 22.8 | 35.7 |
| Others (%) | 7.3 | 6.9 | 12.6 | 11.0 | 18.0 | 18.3 | 23.1 | 17.5 | 7.7 | 6.8 | 12.5 | 11.0 | 17.5 | 19.2 | 23.6 | 17.7 |
| **Donor Age (years)** |  |  |  |  |  |  |  |  |  |  |  |  |  |  |  |  |
| 0-5 (%) | 60.8 | 59.6 | 32.1 | 32.5 | 2.3 | 1.9 | 0.1 | 0.0 | 58.7 | 55.4 | 30.6 | 29.6 | 2.5 | 1.4 | 0.2 | 0.0 |
| 6-12 (%) | 16.5 | 16.2 | 18.7 | 19.2 | 5.4 | 4.0 | 3.3 | 1.0 | 15.5 | 16.0 | 18.8 | 21.0 | 8.0 | 3.6 | 5.4 | 0.9 |
| 13-17 (%) | 9.7 | 9.4 | 14.8 | 11.8 | 10.3 | 8.0 | 7.6 | 5.8 | 11.2 | 12.4 | 18.9 | 16.8 | 17.9 | 12.0 | 13.9 | 8.6 |
| 18-34 (%) | 7.8 | 9.0 | 16.6 | 16.9 | 27.4 | 27.1 | 24.3 | 23.9 | 12.1 | 14.0 | 22.5 | 24.2 | 41.5 | 44.9 | 40.5 | 40.5 |
| 35-49 (%) | 4.3 | 4.4 | 11.0 | 12.9 | 28.3 | 31.1 | 28.4 | 31.5 | 1.7 | 2.0 | 6.3 | 6.3 | 17.6 | 21.9 | 19.9 | 25.1 |
| 50-59 (%) | 0.5 | 1.2 | 4.7 | 5.0 | 16.8 | 17.4 | 19.7 | 21.6 | 0.6 | 0.2 | 2.0 | 1.4 | 8.1 | 11.0 | 11.3 | 14.6 |
| ≥ 60 (%) | 0.2 | 0.2 | 2.0 | 1.7 | 9.6 | 10.5 | 16.6 | 16.1 | 0.1 | 0.0 | 0.9 | 0.5 | 4.5 | 5.2 | 8.8 | 10.3 |
| **Cold ischemia (hours)** |  |  |  |  |  |  |  |  |  |  |  |  |  |  |  |  |
| <8 (%) | 50.4 | 50.8 | 44.9 | 48.9 | 53.3 | 53.4 | 57.2 | 59.8 | 50.5 | 52.6 | 48.4 | 49.0 | 51.8 | 49.8 | 56.3 | 57.2 |
| 8-<12 (%) | 35.7 | 35.5 | 38.5 | 35.2 | 31.3 | 33.2 | 30.5 | 30.8 | 35.7 | 33.2 | 35.5 | 33.2 | 32.4 | 32.8 | 30.5 | 31.4 |
| ≥12 (%) | 13.9 | 13.7 | 16.6 | 15.9 | 15.4 | 13.4 | 12.3 | 9.4 | 13.8 | 14.2 | 16.1 | 17.8 | 15.8 | 17.4 | 13.2 | 11.3 |
| Missing (%) | 8.8 | 7.7 | 9.2 | 9.5 | 8.0 | 8.6 | 7.4 | 6.9 | 8.2 | 8.1 | 9.1 | 10.3 | 7.9 | 7.5 | 7.6 | 7.0 |
| **Transplant year** |  |  |  |  |  |  |  |  |  |  |  |  |  |  |  |  |
| 1988-1994 (%) | 21.0 | 22.4 | 27.2 | 28.8 | 27.6 | 21.0 | 16.9 | 8.1 | 21.0 | 22.6 | 28.1 | 29.3 | 30.6 | 28.2 | 19.8 | 12.5 |
| 1995-1999 (%) | 18.1 | 14.5 | 25.8 | 23.0 | 21.5 | 22.1 | 19.2 | 16.2 | 15.8 | 16.4 | 22.5 | 23.1 | 21.0 | 20.9 | 21.1 | 17.0 |
| 2000-2004 (%) | 18.3 | 18.2 | 21.2 | 22.6 | 18.0 | 19.3 | 21.5 | 22.9 | 21.2 | 16.2 | 22.7 | 19.8 | 15.6 | 17.1 | 19.1 | 22.3 |
| 2005-2009 (%) | 22.9 | 22.5 | 14.0 | 14.2 | 16.2 | 16.6 | 20.7 | 25.3 | 19.9 | 21.0 | 13.8 | 15.3 | 15.7 | 15.9 | 19.1 | 23.8 |
| 2010-2014 (%) | 14.7 | 16.3 | 8.6 | 8.1 | 10.7 | 13.9 | 15.2 | 19.5 | 15.5 | 17.1 | 9.3 | 9.2 | 10.8 | 10.7 | 14.7 | 17.1 |
| 2015-2019 (%) | 4.8 | 6.0 | 3.2 | 3.2 | 5.9 | 7.1 | 6.5 | 8.0 | 6.6 | 6.7 | 3.5 | 3.2 | 6.4 | 7.3 | 6.3 | 7.3 |
| **Donor :recipient weight ratio** |  |  |  |  |  |  |  |  |  |  |  |  |  |  |  |  |
| >=0.9 (%) | 83.8 | 83.7 | 68.7 | 71.1 | 59.6 | 47.4 | 56.6 | 41.3 | 85.4 | 85.0 | 74.4 | 76.5 | 68.7 | 60.9 | 66.1 | 56.9 |
| <0.9 (%) | 16.2 | 16.3 | 31.3 | 28.9 | 40.4 | 52.6 | 43.4 | 58.7 | 14.6 | 15.0 | 25.6 | 23.5 | 31.3 | 39.1 | 33.9 | 43.1 |
| Missing (%) | 8.8 | 8.6 | 10.6 | 10.0 | 10.4 | 7.9 | 6.6 | 3.9 | 8.6 | 9.0 | 9.4 | 10.5 | 9.6 | 8.7 | 6.6 | 4.9 |
| **Graft size** |  |  |  |  |  |  |  |  |  |  |  |  |  |  |  |  |
| Whole (%) | 71.7 | 72.1 | 87.6 | 88.4 | 98.3 | 99.1 | 98.6 | 99.3 | 69.0 | 66.6 | 84.5 | 83.5 | 97.8 | 98.4 | 97.3 | 99.1 |
| Reduced (%) | 28.3 | 27.9 | 12.4 | 11.6 | 1.7 | 0.9 | 1.4 | 0.7 | 31.0 | 33.4 | 15.5 | 16.5 | 2.2 | 1.6 | 2.7 | 0.9 |

Because the unit of analysis was person-time, rather than person, the characteristics presented are weighted by a factor derived from the number of person-years of observation and number of events. For example, 68.4% of the person-years contributed by female recipients between 0–12 years who had a female donor were by people with congenital disease/ biliary atresia as the primary disease.

*SRTR only

**Supplemental Table S3:** Composition of the contrasted experience by recipient sex and age for recipients of female and male donors within the first 3 months (CTS only)

|  | **Female Donor** | | | | | | | | **Male Donor** | | | | | | | |
| --- | --- | --- | --- | --- | --- | --- | --- | --- | --- | --- | --- | --- | --- | --- | --- | --- |
| **Recipient Current Age** | **0-12 years** | | **13-24 years** | | **25-44 years** | | **45+ years** | | **0-12 years** | | **13-24 years** | | **25-44 years** | | **45+ years** | |
| **Recipient sex** | **Female** | **Male** | **Female** | **Male** | **Female** | **Male** | **Female** | **Male** | **Female** | **Male** | **Female** | **Male** | **Female** | **Male** | **Female** | **Male** |
| **Person-years of observation** | 256.8 | 269.2 | 196.4 | 146.2 | 715.9 | 728.8 | 2143.6 | 3137.1 | 367.3 | 384.1 | 193.9 | 198.8 | 602.0 | 1218.7 | 1631.2 | 5326.8 |
| **Deaths** | 93 | 102 | 73 | 63 | 297 | 229 | 889 | 1177 | 130 | 123 | 74 | 58 | 312 | 410 | 786 | 1942 |
| **Expected number of deaths** | 0.33 | 0.45 | 0.05 | 0.08 | 0.53 | 1.08 | 10.24 | 25.42 | 0.44 | 0.56 | 0.05 | 0.13 | 0.45 | 1.92 | 7.65 | 42.71 |
| **Excess mortality rate** | 36.09 | 37.72 | 37.14 | 43.03 | 41.41 | 31.27 | 41.00 | 36.71 | 35.28 | 31.88 | 38.14 | 29.11 | 51.76 | 33.48 | 47.72 | 35.66 |
| **Age at transplant (years)** |  |  |  |  |  |  |  |  |  |  |  |  |  |  |  |  |
| 0-12 (%) | 100.0 | 100.0 | 0.0 | 0.0 | 0.0 | 0.0 | 0.0 | 0.0 | 100.0 | 100.0 | 0.0 | 0.0 | 0.0 | 0.0 | 0.0 | 0.0 |
| 13-24 (%) | 0.0 | 0.0 | 100.0 | 100.0 | 0.0 | 0.0 | 0.0 | 0.0 | 0.0 | 0.0 | 100.0 | 100.0 | 0.0 | 0.0 | 0.0 | 0.0 |
| 25- 44 (%) | 0.0 | 0.0 | 0.0 | 0.0 | 100.0 | 100.0 | 0.0 | 0.0 | 0.0 | 0.0 | 0.0 | 0.0 | 100.0 | 100.0 | 0.0 | 0.0 |
| 45+y (%) | 0.0 | 0.0 | 0.0 | 0.0 | 0.0 | 0.0 | 100.0 | 100.0 | 0.0 | 0.0 | 0.0 | 0.0 | 0.0 | 0.0 | 100.0 | 100.0 |
| **Recipient race** |  |  |  |  |  |  |  |  |  |  |  |  |  |  |  |  |
| White (%) | 85.8 | 86.3 | 88.6 | 94.1 | 92.3 | 91.8 | 95.1 | 94.0 | 86.0 | 86.1 | 91.9 | 92.5 | 92.1 | 92.8 | 94.4 | 94.8 |
| Black (%) | 3.4 | 5.1 | 3.6 | 3.1 | 3.1 | 3.4 | 0.9 | 0.9 | 3.0 | 3.1 | 1.7 | 2.7 | 2.6 | 1.9 | 0.9 | 0.7 |
| Others (%) | 10.9 | 8.6 | 7.8 | 2.8 | 4.6 | 4.8 | 4.0 | 5.1 | 11.0 | 10.8 | 6.4 | 4.8 | 5.2 | 5.3 | 4.7 | 4.4 |
| Missing (%) | 38.3 | 40.1 | 31.7 | 35.5 | 40.6 | 42.8 | 44.2 | 52.5 | 39.7 | 41.8 | 32.6 | 34.1 | 36.4 | 41.5 | 39.7 | 49.8 |
| **Primary disease** |  |  |  |  |  |  |  |  |  |  |  |  |  |  |  |  |
| Congenital/biliary atresia (%) | 53.9 | 44.6 | 10.2 | 12.9 | 4.2 | 1.7 | 2.4 | 0.4 | 54.8 | 47.7 | 10.8 | 11.7 | 5.5 | 1.3 | 3.4 | 0.4 |
| Alcohol Associated Liver Disease (%) | 0.1 | 0.1 | 0.1 | 0.3 | 7.3 | 14.3 | 13.2 | 21.5 | 0.0 | 0.1 | 0.0 | 0.1 | 7.1 | 15.3 | 12.8 | 22.5 |
| Liver tumors (%) | 5.0 | 5.7 | 3.5 | 4.2 | 6.0 | 7.8 | 9.3 | 20.8 | 4.8 | 7.4 | 2.7 | 4.9 | 6.0 | 7.3 | 8.2 | 21.0 |
| Metabolic liver disease (%) | 11.3 | 15.7 | 13.1 | 14.3 | 5.1 | 4.6 | 1.6 | 2.2 | 11.7 | 14.9 | 16.5 | 13.8 | 5.9 | 5.0 | 1.9 | 2.2 |
| Fulminant liver failure (%) | 10.4 | 12.5 | 33.7 | 21.4 | 19.1 | 9.4 | 6.5 | 2.5 | 9.2 | 9.0 | 29.8 | 20.3 | 21.1 | 7.4 | 7.5 | 1.9 |
| Autoimmune conditions (%) | 2.1 | 2.7 | 18.4 | 21.3 | 26.7 | 18.3 | 27.9 | 6.4 | 2.7 | 2.3 | 17.2 | 21.6 | 23.8 | 15.8 | 25.0 | 5.5 |
| Hepatitis C (%) | 1.9 | 2.7 | 2.3 | 7.1 | 11.9 | 25.8 | 25.1 | 33.5 | 2.1 | 2.7 | 3.9 | 6.4 | 12.1 | 29.2 | 26.0 | 33.3 |
| Others (%) | 15.3 | 16.0 | 18.8 | 18.5 | 19.7 | 18.1 | 14.0 | 12.8 | 14.7 | 15.8 | 19.1 | 21.2 | 18.6 | 18.6 | 15.3 | 13.3 |
| **Donor Age (years)** |  |  |  |  |  |  |  |  |  |  |  |  |  |  |  |  |
| 0-5 (%) | 18.3 | 17.0 | 0.6 | 0.7 | 0.1 | 0.0 | 0.1 | 0.1 | 20.6 | 19.6 | 1.5 | 1.2 | 0.1 | 0.1 | 0.2 | 0.1 |
| 6-12 (%) | 17.1 | 17.0 | 4.3 | 6.4 | 1.6 | 0.5 | 0.9 | 0.3 | 20.8 | 19.2 | 9.8 | 7.5 | 2.8 | 0.7 | 1.7 | 0.2 |
| 13-17 (%) | 11.7 | 11.9 | 9.8 | 10.3 | 4.7 | 3.6 | 3.2 | 1.8 | 11.9 | 13.0 | 17.0 | 15.2 | 9.2 | 5.4 | 6.9 | 3.2 |
| 18-34 (%) | 23.6 | 22.7 | 22.4 | 21.2 | 21.4 | 18.4 | 14.9 | 11.9 | 30.4 | 31.0 | 33.6 | 33.2 | 36.8 | 33.6 | 29.4 | 22.3 |
| 35-49 (%) | 22.5 | 23.8 | 34.5 | 34.3 | 32.1 | 33.3 | 26.4 | 24.8 | 13.7 | 13.9 | 22.4 | 23.7 | 24.1 | 27.3 | 23.0 | 24.2 |
| 50-59 (%) | 5.3 | 5.8 | 17.9 | 18.8 | 21.1 | 22.5 | 22.3 | 22.9 | 2.3 | 2.8 | 10.8 | 12.1 | 15.5 | 18.1 | 16.5 | 20.1 |
| ≥ 60 (%) | 1.5 | 1.8 | 10.5 | 8.3 | 19.0 | 21.6 | 32.2 | 38.2 | 0.3 | 0.5 | 4.8 | 7.2 | 11.4 | 14.7 | 22.3 | 29.8 |
| **Cold ischemia (hours)** |  |  |  |  |  |  |  |  |  |  |  |  |  |  |  |  |
| <8 (%) | 39.9 | 39.3 | 42.7 | 39.8 | 43.9 | 41.4 | 46.5 | 47.8 | 38.8 | 39.0 | 42.6 | 40.7 | 44.4 | 41.7 | 46.2 | 47.7 |
| 8-<12 (%) | 38.3 | 40.4 | 37.7 | 37.7 | 36.4 | 38.2 | 35.6 | 35.5 | 40.7 | 38.6 | 36.3 | 35.6 | 34.2 | 35.5 | 34.5 | 35.8 |
| ≥12 (%) | 21.8 | 20.3 | 19.6 | 22.5 | 19.7 | 20.4 | 18.0 | 16.7 | 20.5 | 22.4 | 21.2 | 23.7 | 21.5 | 22.9 | 19.3 | 16.6 |
| Missing (%) | 26.7 | 30.9 | 29.1 | 24.0 | 28.1 | 25.3 | 25.6 | 25.7 | 31.2 | 31.3 | 26.2 | 27.2 | 26.2 | 27.0 | 26.2 | 26.0 |
| **Transplant year** |  |  |  |  |  |  |  |  |  |  |  |  |  |  |  |  |
| 1988-1994 (%) | 15.7 | 12.1 | 16.3 | 11.1 | 13.2 | 13.3 | 8.7 | 5.1 | 17.2 | 17.0 | 21.2 | 16.4 | 19.7 | 15.8 | 13.3 | 6.8 |
| 1995-1999 (%) | 15.6 | 15.4 | 15.1 | 18.2 | 16.8 | 17.9 | 14.1 | 11.4 | 15.8 | 17.3 | 20.4 | 21.8 | 20.8 | 20.3 | 16.4 | 12.7 |
| 2000-2004 (%) | 16.4 | 17.4 | 19.5 | 19.1 | 20.1 | 20.3 | 18.0 | 17.4 | 18.4 | 17.4 | 18.9 | 17.8 | 16.6 | 21.7 | 20.4 | 18.0 |
| 2005-2009 (%) | 19.1 | 21.1 | 16.8 | 16.1 | 19.3 | 19.1 | 20.5 | 22.9 | 18.3 | 18.2 | 14.1 | 16.9 | 16.9 | 17.8 | 17.7 | 21.1 |
| 2010-2014 (%) | 18.9 | 18.7 | 18.0 | 19.9 | 16.9 | 16.5 | 21.4 | 24.3 | 16.2 | 16.4 | 13.5 | 14.5 | 15.1 | 13.1 | 17.6 | 22.3 |
| 2015-2019 (%) | 14.3 | 15.4 | 14.3 | 15.6 | 13.8 | 12.9 | 17.4 | 18.8 | 14.1 | 13.6 | 11.9 | 12.6 | 10.8 | 11.2 | 14.7 | 19.1 |
| **Graft size** |  |  |  |  |  |  |  |  |  |  |  |  |  |  |  |  |
| Whole (%) | 38.1 | 35.3 | 89.4 | 88.8 | 94.7 | 96.7 | 95.6 | 97.7 | 37.4 | 38.0 | 86.4 | 87.4 | 91.5 | 95.8 | 92.6 | 96.9 |
| Reduced (%) | 61.9 | 64.7 | 10.6 | 11.2 | 5.3 | 3.3 | 4.4 | 2.3 | 62.6 | 62.0 | 13.6 | 12.6 | 8.5 | 4.2 | 7.4 | 3.1 |

Because the unit of analysis was person-time, rather than person, the characteristics presented are weighted by a factor derived from the number of person-years of observation and number of events. For example, 53.9% of the person-years contributed by female recipients between 0–12 years who had a female donor were by people with congenital disease/ biliary atresia as the primary disease.

*SRTR only

**Supplemental Table S4:** Composition of the contrasted experience by recipient sex and age for recipients of female and male donors after the first 3 months (CTS only)

|  | **Female Donor** | | | | | | | | **Male Donor** | | | | | | | |
| --- | --- | --- | --- | --- | --- | --- | --- | --- | --- | --- | --- | --- | --- | --- | --- | --- |
| **Recipient Current Age** | **0-12 years** | | **13-24 years** | | **25-44 years** | | **45+ years** | | **0-12 years** | | **13-24 years** | | **25-44 years** | | **45+ years** | |
| **Recipient sex** | **Female** | **Male** | **Female** | **Male** | **Female** | **Male** | **Female** | **Male** | **Female** | **Male** | **Female** | **Male** | **Female** | **Male** | **Female** | **Male** |
| **Person-years of observation** | 5499.9 | 5687.9 | 5099.1 | 4662.6 | 15863.5 | 13443.1 | 64295.1 | 79292.7 | 8151.1 | 8276.2 | 6192.2 | 6631.0 | 14597.8 | 22033.4 | 52192.9 | 140325.9 |
| **Deaths** | 49 | 63 | 69 | 56 | 334 | 404 | 2606 | 3773 | 63 | 95 | 68 | 84 | 302 | 677 | 2002 | 6606 |
| **Expected number of deaths** | 1.5 | 1.9 | 1.1 | 2.3 | 10.9 | 18.4 | 474.5 | 851.6 | 2.2 | 2.6 | 1.3 | 3.4 | 10.2 | 31.8 | 372.9 | 1530.6 |
| **Excess mortality rate** | 0.86 | 1.07 | 1.33 | 1.15 | 2.04 | 2.87 | 3.32 | 3.68 | 0.75 | 1.12 | 1.08 | 1.22 | 2.00 | 2.93 | 3.12 | 3.62 |
| **Age at transplant (years)** |  |  |  |  |  |  |  |  |  |  |  |  |  |  |  |  |
| 0-12 (%) | 100.0 | 100.0 | 45.1 | 51.0 | 1.3 | 1.5 | 0.0 | 0.0 | 100.0 | 100.0 | 51.3 | 56.2 | 2.0 | 1.4 | 0.0 | 0.0 |
| 13-24 (%) | 0.0 | 0.0 | 54.9 | 49.0 | 19.2 | 13.6 | 0.0 | 0.0 | 0.0 | 0.0 | 48.7 | 43.8 | 22.1 | 12.4 | 0.1 | 0.0 |
| 25- 44 (%) | 0.0 | 0.0 | 0.0 | 0.0 | 79.5 | 84.9 | 13.9 | 11.6 | 0.0 | 0.0 | 0.0 | 0.0 | 75.9 | 86.1 | 15.0 | 12.1 |
| 45+y (%) | 0.0 | 0.0 | 0.0 | 0.0 | 0.0 | 0.0 | 86.1 | 88.4 | 0.0 | 0.0 | 0.0 | 0.0 | 0.0 | 0.0 | 84.9 | 87.9 |
| **Recipient race** |  |  |  |  |  |  |  |  |  |  |  |  |  |  |  |  |
| White (%) | 86.1 | 87.7 | 88.3 | 92.4 | 91.8 | 92.1 | 95.5 | 93.7 | 87.3 | 86.9 | 91.2 | 89.9 | 92.7 | 92.9 | 95.5 | 95.4 |
| Black (%) | 3.9 | 4.9 | 2.9 | 3.0 | 2.9 | 3.6 | 0.9 | 0.9 | 3.1 | 3.5 | 1.7 | 2.1 | 2.4 | 2.1 | 0.8 | 0.8 |
| Others (%) | 10.0 | 7.4 | 8.8 | 4.6 | 5.3 | 4.3 | 3.6 | 5.4 | 9.6 | 9.6 | 7.0 | 8.0 | 5.0 | 5.0 | 3.7 | 3.8 |
| Missing (%) | 33.6 | 37.6 | 28.9 | 30.4 | 33.5 | 38.1 | 37.6 | 47.3 | 38.5 | 38.6 | 30.9 | 31.4 | 32.7 | 36.9 | 35.4 | 44.8 |
| **Primary disease** |  |  |  |  |  |  |  |  |  |  |  |  |  |  |  |  |
| Congenital/biliary atresia (%) | 61.0 | 51.4 | 26.7 | 28.2 | 5.1 | 3.9 | 2.3 | 0.4 | 61.2 | 53.8 | 31.3 | 31.9 | 6.3 | 2.6 | 3.5 | 0.4 |
| Alcohol Associated Liver Disease (%) | 0.1 | 0.2 | 0.2 | 0.0 | 4.0 | 9.7 | 11.3 | 20.4 | 0.0 | 0.1 | 0.0 | 0.1 | 3.8 | 10.4 | 10.5 | 21.8 |
| Liver tumors (%) | 3.8 | 4.5 | 3.1 | 3.7 | 4.3 | 5.7 | 6.3 | 17.2 | 3.9 | 5.4 | 2.2 | 4.1 | 4.2 | 5.7 | 5.8 | 16.9 |
| Metabolic liver disease (%) | 9.3 | 14.4 | 15.7 | 16.1 | 7.8 | 7.2 | 1.6 | 2.4 | 9.9 | 14.2 | 18.3 | 15.1 | 9.3 | 8.1 | 1.9 | 2.6 |
| Fulminant liver failure (%) | 8.1 | 10.5 | 22.4 | 16.8 | 24.8 | 14.4 | 8.2 | 2.7 | 7.9 | 7.7 | 19.1 | 14.3 | 27.8 | 11.1 | 8.7 | 2.0 |
| Autoimmune conditions (%) | 1.4 | 1.9 | 9.1 | 11.6 | 23.7 | 19.8 | 34.7 | 7.7 | 2.1 | 1.9 | 8.2 | 10.3 | 19.7 | 18.4 | 31.2 | 7.2 |
| Hepatitis C (%) | 1.2 | 1.9 | 2.1 | 4.5 | 8.4 | 20.4 | 21.0 | 34.7 | 2.1 | 2.4 | 3.1 | 5.0 | 8.6 | 22.8 | 22.2 | 33.9 |
| Others (%) | 15.3 | 15.1 | 20.6 | 19.0 | 21.8 | 19.0 | 14.6 | 14.5 | 12.9 | 14.6 | 17.8 | 19.2 | 20.2 | 20.9 | 16.1 | 15.1 |
| **Donor Age (years)** |  |  |  |  |  |  |  |  |  |  |  |  |  |  |  |  |
| 0-5 (%) | 23.1 | 21.0 | 8.2 | 10.4 | 0.3 | 0.3 | 0.1 | 0.1 | 23.0 | 21.6 | 9.8 | 9.6 | 0.7 | 0.3 | 0.1 | 0.1 |
| 6-12 (%) | 17.5 | 17.1 | 16.5 | 17.9 | 3.3 | 1.6 | 1.4 | 0.4 | 21.0 | 19.8 | 20.9 | 23.3 | 4.5 | 1.9 | 2.3 | 0.4 |
| 13-17 (%) | 12.4 | 11.2 | 13.7 | 13.6 | 6.5 | 5.1 | 4.5 | 2.5 | 12.0 | 13.0 | 17.2 | 16.2 | 11.6 | 7.4 | 8.8 | 4.3 |
| 18-34 (%) | 20.7 | 21.9 | 20.6 | 19.2 | 25.7 | 22.3 | 19.4 | 15.2 | 29.6 | 29.9 | 30.4 | 27.5 | 40.2 | 38.5 | 36.9 | 28.2 |
| 35-49 (%) | 20.1 | 21.5 | 25.4 | 23.8 | 32.5 | 34.8 | 31.3 | 27.9 | 12.1 | 13.1 | 14.2 | 15.8 | 22.2 | 26.8 | 23.0 | 26.0 |
| 50-59 (%) | 4.9 | 6.1 | 10.5 | 11.0 | 18.4 | 20.5 | 21.3 | 23.4 | 2.1 | 1.9 | 6.0 | 5.1 | 13.3 | 15.2 | 14.6 | 18.7 |
| ≥ 60 (%) | 1.3 | 1.2 | 5.0 | 4.1 | 13.3 | 15.5 | 22.0 | 30.6 | 0.1 | 0.7 | 1.5 | 2.5 | 7.5 | 9.8 | 14.3 | 22.3 |
| **Cold ischemia (hours)** |  |  |  |  |  |  |  |  |  |  |  |  |  |  |  |  |
| <8 (%) | 38.3 | 36.1 | 36.7 | 36.9 | 40.6 | 39.1 | 41.6 | 43.4 | 37.2 | 36.5 | 34.2 | 34.4 | 40.3 | 39.8 | 41.4 | 42.9 |
| 8-<12 (%) | 38.4 | 42.1 | 37.7 | 37.1 | 37.2 | 38.6 | 36.6 | 37.0 | 40.7 | 39.4 | 40.1 | 37.0 | 36.4 | 35.1 | 35.0 | 36.9 |
| ≥12 (%) | 23.3 | 21.8 | 25.6 | 25.9 | 22.2 | 22.3 | 21.8 | 19.6 | 22.1 | 24.1 | 25.7 | 28.5 | 23.3 | 25.0 | 23.6 | 20.3 |
| Missing (%) | 25.3 | 32.1 | 28.5 | 28.9 | 27.9 | 24.9 | 23.9 | 23.4 | 31.9 | 33.5 | 31.4 | 30.7 | 26.7 | 27.6 | 25.9 | 24.2 |
| **Transplant year** |  |  |  |  |  |  |  |  |  |  |  |  |  |  |  |  |
| 1988-1994 (%) | 18.3 | 14.5 | 22.0 | 18.7 | 17.0 | 17.4 | 15.1 | 8.4 | 21.5 | 18.8 | 27.5 | 23.1 | 24.4 | 19.6 | 20.4 | 10.5 |
| 1995-1999 (%) | 20.6 | 19.6 | 23.9 | 28.5 | 23.5 | 22.2 | 21.9 | 17.7 | 18.5 | 21.6 | 26.8 | 30.2 | 26.5 | 25.3 | 24.0 | 19.8 |
| 2000-2004 (%) | 20.5 | 21.5 | 22.6 | 23.1 | 23.7 | 23.9 | 24.8 | 25.0 | 22.4 | 20.7 | 21.9 | 21.5 | 19.2 | 23.2 | 24.1 | 25.7 |
| 2005-2009 (%) | 20.7 | 24.4 | 17.7 | 15.2 | 19.6 | 18.7 | 20.5 | 26.4 | 20.1 | 21.3 | 13.2 | 15.4 | 17.2 | 18.2 | 17.4 | 23.7 |
| 2010-2014 (%) | 15.2 | 15.7 | 10.3 | 10.7 | 12.5 | 13.6 | 13.8 | 17.5 | 13.2 | 13.6 | 7.9 | 7.6 | 9.9 | 10.1 | 10.9 | 15.4 |
| 2015-2019 (%) | 4.8 | 4.4 | 3.5 | 3.7 | 3.7 | 4.2 | 3.9 | 4.9 | 4.3 | 3.9 | 2.7 | 2.2 | 2.8 | 3.5 | 3.1 | 4.8 |
| **Graft size** |  |  |  |  |  |  |  |  |  |  |  |  |  |  |  |  |
| Whole (%) | 36.6 | 33.2 | 74.3 | 70.7 | 94.4 | 96.1 | 95.7 | 97.5 | 36.0 | 36.5 | 65.2 | 65.0 | 90.9 | 94.8 | 92.6 | 96.7 |
| Reduced (%) | 63.4 | 66.8 | 25.7 | 29.3 | 5.6 | 3.9 | 4.3 | 2.5 | 64.0 | 63.5 | 34.8 | 35.0 | 9.1 | 5.2 | 7.4 | 3.3 |

Because the unit of analysis was person-time, rather than person, the characteristics presented are weighted by a factor derived from the number of person-years of observation and number of events. For example, 61.0 % of the person-years contributed by female recipients between 0–12 years who had a female donor were by people with congenital disease/ biliary atresia as the primary disease.

*SRTR only

**Supplemental Table S5: Condition at time of transplant by recipient sex and age**

| **Cohort** | **Severity measure** |  | **Female donor** | | **Male donor** | |
| --- | --- | --- | --- | --- | --- | --- |
|  |  | **Recipient age at transplant** | **Female recipient** | **Male recipient** | **Female recipient** | **Male recipient** |
| **SRTR** | **In ICU at time of transplant** | **0-12y (%)** | 469 (22.5) | 497 (25.1) | 669 (22.5) | 748 (25.5) |
|  |  | **13-24y (%)** | 418 (38.7) | 252 (31.3) | 567 (38.8) | 373 (30.0) |
|  |  | **25-44y (%)** | 1002 (25.0) | 827 (18.6) | 1393 (29.6) | 1535 (17.6) |
|  |  | **>=45y (%)** | 2239 (13.4) | 2721 (11.1) | 3001 (16.7) | 4708 (10.6) |
|  |  |  |  |  |  |  |
|  | **On life support at time of transplant** | **0-12y (%)** | 216 (10.4) | 245 (12.4) | 332 (11.2) | 365 (12.5) |
|  |  | **13-24y (%)** | 273 (25.3) | 156 (19.4) | 345 (23.4) | 220 (17.7) |
|  |  | **25-44y (%)** | 634 (15.9) | 462 (10.4) | 846 (18.0) | 839 (9.6) |
|  |  | **>=45y (%)** | 1187 (7.1) | 1313 (5.3) | 1622 (9.1) | 2424 (5.5) |
|  |  |  |  |  |  |  |
| **CTS** | **Urgent or super-urgent** | **0-12y (%)** | 317 (29.7) | 377 (34.0) | 410 (27.5) | 429 (28.1) |
|  |  | **13-24y (%)** | 403 (46.5) | 235 (37.2) | 389 (45.2) | 267 (31.6) |
|  |  | **25-44y (%)** | 992 (31.0) | 600 (19.3) | 846 (31.5) | 1017 (19.7) |
|  |  | **>=45y (%)** | 1395 (15.1) | 1305 (9.80) | 1247 (17.7) | 2153 (9.60) |

**Supplemental Table S6: Sensitivity Analysis Examining the association between Recipient Sex and Excess Mortality in Multivariable Models Including and Excluding Donor: Recipient Weight Ratio (SRTR only)**

|  | **With Donor:Recipient**  **Weight Ratio**  **RER (95% CI)** | **Without Donor:Recipient**  **Weight Ratio**  **RER (95% CI)** |
| --- | --- | --- |
| **First 3 Months**  **Female Donor**  Recipient Age  0-12  13-24  25-44  45+  **Male Donor**  Recipient Age  0-12  13-24  25-44  45+ | 0.98 (0.77-1.26)  1.04 (0.72-1.52)  1.30 (1.07-1.58)  1.22 (1.11-1.33)  0.94 (0.75-1.18)  1.30 (0.94-1.80)  1.54 (1.03-1.81)  1.43 (1.32-1.55) | 0.98 (0.77-1.26)  1.05 (0.72-1.53)  1.26 (1.04-1.53)  1.17 (1.08-1.28)  0.94 (0.75-1.17)  1.30 (0.94-1.79)  1.47 (1.25-1.73)  1.36 (1.26-1.47) |
| **After 3 Months**  **Female Donor**  Recipient Age  0-12  13-24  25-44  45+  **Male Donor**  Recipient Age  0-12  13-24  25-44  45+ | 0.77 (0.58-1.02)  1.17 (0.92-1.50)  0.89 (0.79-1.00)  0.99 (0.95-1.04)  0.85 (0.68-1.07)  1.27 (1.03-1.56)  1.13 (1.02-1.24)  1.07 (1.03-1.12) | 0.78 (0.60-1.01)  1.13 (0.90-1.42)  0.85 (0.76-0.95)  0.91 (0.88-0.95)  0.86 (0.69-1.07)  1.19 (0.98-1.45)  1.03 (0.94-1.13)  0.97 (0.95-1.00) |

Relative excess risks (RER) show the excess mortality risk in female recipients compared with male recipients. Models were adjusted for transplant year, primary disease (congenital/biliary atresia, alcohol related liver disease, liver tumors, metabolic liver disease, fulminant liver failure, autoimmune conditions, hepatitis C), donor age (quadratic), recipient race (White, Black, Other), cold ischemia time, and graft size (whole vs. other). Results in the first column also include donor:recipient weight ratio. Relative Excess Risks (RER) are analogous to hazard ratios and interpreted as excess risk of mortality associated with transplant in female recipients, relative to that in males.

**Supplemental Table S7: Sensitivity Analysis Examining the association between Recipient Sex and Excess Mortality in the primary analysis compared with an analysis including data extended to 2024 (SRTR only)**

|  | **Primary analysis (Data to 2019)**  **RER (95% CI)** | **With Data Extended to 2024**  **RER (95% CI)** |
| --- | --- | --- |
| **First 3 Months**  **Female Donor**  Recipient Age  0-12  13-24  25-44  45+  **Male Donor**  Recipient Age  0-12  13-24  25-44  45+ | 0.98 (0.77-1.26)  1.04 (0.72-1.52)  **1.30 (1.07-1.58)**  **1.22 (1.11-1.33)**  0.94 (0.75-1.18)  1.30 (0.94-1.80)  **1.54 (1.03-1.81)**  **1.43 (1.32-1.55)** | 1.00 (0.77-1.31)  1.06 (0.73-1.55)  **1.37 (1.14-1.66)**  **1.23 (1.08-1.28)**  1.03 (0.81-1.29)  **1.47 (1.02-2.11)**  **1.68 (1.44-1.96)**  **1.42 (1.32-1.53)** |
| **After 3 Months**  **Female Donor**  Recipient Age  0-12  13-24  25-44  45+  **Male Donor**  Recipient Age  0-12  13-24  25-44  45+ | 0.77 (0.58-1.02)  1.17 (0.92-1.50)  0.89 (0.79-1.00)  0.99 (0.95-1.04)  0.85 (0.68-1.07)  **1.27 (1.03-1.56)**  **1.13 (1.02-1.24)**  **1.07 (1.03-1.12)** | 0.75 (0.56-1.00)  1.20 (0.93-1.54)  0.96 (0.85-1.08)  1.01 (0.97-1.06)  0.79 (0.62-1.00)  1.18 (0.94-1.47)  **1.18 (1.07-1.30)**  **1.32 (1.24-1.41)** |

Relative excess risks (RER) show the excess mortality risk in female recipients compared with male recipients. Models were adjusted for transplant year, primary disease (congenital/biliary atresia, alcohol related liver disease, liver tumors, metabolic liver disease, fulminant liver failure, autoimmune conditions, hepatitis C), donor age (quadratic), recipient race (White, Black, Other), cold ischemia time, graft size (whole vs. other), and donor:recipient weight ratio. Results in the second column include data through to 2024. Relative Excess Risks (RER) are analogous to hazard ratios and interpreted as excess risk of mortality associated with transplant in female recipients, relative to that in males.

**Supplemental Table S8a: Causes of Death by Recipient Sex and Age in SRTR**

|  | **Female Donor** | | | | | | | | **Male Donor** | | | | | | | |
| --- | --- | --- | --- | --- | --- | --- | --- | --- | --- | --- | --- | --- | --- | --- | --- | --- |
| **Recipient Current Age** | **0-12 years** | | **13-24 years** | | **25-44 years** | | **45+ years** | | **0-12 years** | | **13-24 years** | | **25-44 years** | | **45+ years** | |
| **Recipient sex** | **Female** | **Male** | **Female** | **Male** | **Female** | **Male** | **Female** | **Male** | **Female** | **Male** | **Female** | **Male** | **Female** | **Male** | **Female** | **Male** |
| Cardiovascular (%) | 15.1 | 15.9 | 14.7 | 16.8 | 13.8 | 14.6 | 17.2 | 16.8 | 17.0 | 14.7 | 13.4 | 15.6 | 14.6 | 14.2 | 17.5 | 17.9 |
| Infection (%) | 18.8 | 18.4 | 13.5 | 16.8 | 20.0 | 20.0 | 16.4 | 14.7 | 17.7 | 19.9 | 15.4 | 15.1 | 20.1 | 19.3 | 16.4 | 13.3 |
| Malignancy (%) | 10.9 | 11.8 | 4.6 | 9.6 | 7.8 | 10.2 | 15.7 | 21.3 | 11.6 | 15.3 | 6.2 | 10.5 | 7.5 | 10.7 | 15.1 | 22.0 |
| Other (%) | 36.0 | 40.4 | 46.2 | 41.3 | 37.6 | 33.9 | 39.9 | 37.2 | 37.3 | 36.0 | 43.8 | 44.1 | 37.5 | 34.0 | 39.6 | 36.8 |
| Graft failure (%) | 19.3 | 13.5 | 21.0 | 15.6 | 20.8 | 21.3 | 10.8 | 10.0 | 16.4 | 14.1 | 21.2 | 14.7 | 20.3 | 21.8 | 11.4 | 10.1 |
| Unknown/missing (%) | 2.5 | 6.5 | 8.1 | 8.7 | 12.6 | 11.8 | 23.6 | 21.1 | 6.0 | 6.2 | 10.5 | 7.4 | 11.2 | 12.8 | 22.0 | 21.9 |

In total there were 59,081 deaths, of which 88% occurred at ≥45 years of age. Cause of death was missing in 20.6% overall.

**Supplemental Table S8b: Causes of Death by Recipient Sex and Age in CTS**

|  | **Female Donor** | | | | | | | | **Male Donor** | | | | | | | |
| --- | --- | --- | --- | --- | --- | --- | --- | --- | --- | --- | --- | --- | --- | --- | --- | --- |
| **Recipient Current Age** | **0-12 years** | | **13-24 years** | | **25-44 years** | | **45+ years** | | **0-12 years** | | **13-24 years** | | **25-44 years** | | **45+ years** | |
| **Recipient sex** | **Female** | **Male** | **Female** | **Male** | **Female** | **Male** | **Female** | **Male** | **Female** | **Male** | **Female** | **Male** | **Female** | **Male** | **Female** | **Male** |
| Cardiovascular (%) | 12.0 | 13.3 | 9.1 | 9.2 | 13.1 | 9.2 | 12.6 | 11.2 | 13.0 | 5.9 | 9.7 | 11.5 | 12.6 | 9.9 | 11.7 | 11.2 |
| Infection (%) | 15.5 | 17.0 | 23.8 | 21.0 | 22.9 | 26.6 | 25.0 | 23.7 | 19.3 | 18.7 | 22.1 | 23.7 | 20.9 | 26.7 | 25.2 | 22.7 |
| Malignancy (%) | 7.0 | 6.7 | 7.7 | 6.7 | 11.4 | 13.3 | 13.4 | 20.5 | 5.7 | 8.2 | 4.8 | 7.2 | 10.6 | 12.0 | 14.6 | 21.2 |
| Other (%) | 29.6 | 31.5 | 28.0 | 35.3 | 25.9 | 24.7 | 24.1 | 22.1 | 36.5 | 32.9 | 28.3 | 24.5 | 27.2 | 24.6 | 23.1 | 22.0 |
| Graft failure (%) | 29.6 | 21.8 | 27.3 | 21.8 | 17.4 | 16.6 | 13.4 | 11.6 | 20.8 | 24.7 | 24.8 | 20.1 | 21.9 | 16.9 | 15.0 | 11.9 |
| Unknown/missing (%) | 6.3 | 9.7 | 4.2 | 5.9 | 9.2 | 9.5 | 11.5 | 11.0 | 4.7 | 9.6 | 10.3 | 12.9 | 6.9 | 9.8 | 10.4 | 11.0 |

**Supplemental Figure S1a: Unadjusted IPD-meta analysis results for excess mortality in female versus male recipients of female and male donors during the first 3 months post-transplant.**

Forest plots show the relative excess risks (RER) of mortality in female versus male recipients in each age interval. RER are shown separately for the SRTR and CTS cohorts, as well as the pooled RER from the meta-analysis. Models are unadjusted. RER are analogous to hazard ratios and interpreted as excess risk of mortality associated with transplant in female recipients, relative to that in males.

**Supplemental Figure S1b: Unadjusted IPD-meta analysis results for excess mortality in female versus male recipients of female and male donors after the first 3 months post-transplant.**

Forest plots show the relative excess risks (RER) of mortality in female versus male recipients in each age interval. RER are shown separately for the SRTR and CTS cohorts, as well as the pooled RER from the meta-analysis. Models are unadjusted. RER are analogous to hazard ratios and interpreted as excess risk of mortality associated with transplant in female recipients, relative to that in males.

**Supplemental Figure S2:** Summary of Adjusted Relative Excess Risks of Mortality and Adjusted Relative Hazards of Graft Failure in Female versus Male Recipients in SRTR in (a) the first 3 months post-transplant and (b) after the first 3 months post-transplant

b)

a)

HR are interpreted as the hazards of graft failure in females relative to the hazard in males. SRTR-specific RER shown here are analogous to hazard ratios and interpreted as excess risk of mortality associated with transplant in female recipients, relative to that in males.

**REFERENCES**

1.	Foster, B.J., et al., *Changes in Excess Mortality from End Stage Renal Disease in the United States from 1995 to 2013.* Clin J Am Soc Nephrol, 2018. **13**(1): p. 91-99.

2.	Dickman, P.W., et al., *Regression models for relative survival.* Stat Med, 2004. **23**(1): p. 51-64.
